# Code-Based Versus AutoML Methods for Pill Recognition in Clinical Settings: Comparative Performance Study

**DOI:** 10.2196/79160

**Published:** 2026-04-10

**Authors:** Amir Reza Ashraf, Richárd Rádli, Zsolt Vörösházi, András Fittler

**Affiliations:** 1 Department of Pharmaceutics, Faculty of Pharmacy, University of Pécs, Rókus utca 4, Pécs, H-7624, Hungary, +36 72503650 ext 28841; 2 Image Processing Research Laboratory, University of Pannonia, Veszprém, Hungary

**Keywords:** pill recognition, clinical pharmacy, medication safety, medication errors, clinical decision support, You Only Look Once, YOLO11, automated machine learning, AutoML, computer vision, object detection, deep learning

## Abstract

**Background:**

Visual identification and verification of medications during dispensing and administration are prone to human error, particularly in high-pressure and high-volume clinical settings. Misidentification can lead to medication errors, posing risks to patient safety and placing a burden on health care systems. Recent advances in computer vision and object detection offer promising solutions for automated solid oral dosage form (pill) recognition. However, comprehensive studies comparing code-based and no-code (automated machine learning [AutoML]) approaches for pill recognition are lacking.

**Objective:**

This study aimed to evaluate and compare the performance, cost, usability, and deployment feasibility of pill recognition models developed with Ultralytics YOLO11 and 3 cloud-based AutoML platforms (Amazon Rekognition Custom Labels, Google Vertex artificial intelligence [AI] AutoML Vision, and Microsoft Azure Custom Vision) using multiple datasets, including real-world clinical images.

**Methods:**

Five training subsets of increasing size (1230, 3450, 7380, 14,400, and 26,880 images) from 30 commonly dispensed medications were used to train models on YOLO11 and 3 AutoML platforms. Models were evaluated on 6 datasets from different environments: clinical images from 3 hospitals, a verification dataset, a laboratory dataset, and an exhaustive testing set. Performance metrics, including accuracy, precision, recall, and mean average precision, were calculated. We evaluated the impact of training data size on performance and benchmarked training time, platform costs, and limitations.

**Results:**

No single platform dominated across all test environments. On the verification dataset (optimal conditions), accuracy ranged from 80.83% (YOLO11) to 91.60% (Google Vertex AI) when trained with the full training dataset. YOLO11 showed consistent performance improvement with increasing training data (accuracy: 63.06%-80.83%) and achieved near-perfect precision and mean average precision scores (0.95‐1.00). Google Vertex AI reached above 90% accuracy on 3 training subsets but showed unpredictable declines. Amazon Rekognition maintained near-perfect precision (0.92‐1.00) but had the highest false negative rates (up to 0.74), missing many pills. Custom Vision demonstrated steady performance improvements (77.08%-85.62% accuracy) but lagged behind other AutoML platforms, probably due to its older YOLOv2-based architecture. On clinical datasets, accuracy fluctuated (20.62%-90%) depending on the dataset and platform. Training costs and time varied: YOLO11 (open-source), Microsoft Azure (US $9.50-US $28.60, allowed user-predefined training duration), Google Vertex AI (US $69.30 with consistent 2.5‐3-hour training times), and Amazon Rekognition (US $5.43-US $43.89 with size-dependent training time scaling, reaching nearly 40 hours on the full 26,880-image dataset).

**Conclusions:**

Each platform offers distinct advantages and trade-offs: YOLO11 provides the highest flexibility and lowest platform costs but requires technical expertise, while AutoML platforms can offer high performance at a higher cost but with limited user control, introducing unpredictability. The performance variations demonstrate that successful clinical deployment requires careful platform selection based on specific performance requirements, budget constraints, and available technical resources, followed by rigorous validation using real-world, representative data to ensure patient safety in clinical workflows.

## Introduction

### Background

Medication errors are preventable failures in medical care that can potentially lead to patient harm and increased health care costs, with a global financial burden estimated at US $42 billion annually [1]. These errors are responsible for an estimated 7000‐9000 deaths per year in the United States [1] and contribute to 1708 deaths in England [2], highlighting the urgent need for improved medication safety strategies. Medication errors can occur at various stages of therapy, from prescribing to dispensing and administration of medications [3], and are among the most common causes of death [4]. Medication therapy management in hospitals, clinics, and inpatient health care settings is often managed under the supervision of a clinical pharmacist, who is responsible for monitoring patients’ treatment process and identifying any discrepancies [5]. During inpatient care, medication reconciliation, that is, the process in which pharmacists compare the medications a patient is currently taking or should be taking with newly ordered therapies, often requires visually distinguishing between numerous solid oral dosage forms (eg, pills, tablets, and capsules, often referred to collectively as “pills”) based on the patient’s therapy regimen. This process can be challenging because these products often show only subtle differences in their physical characteristics [6]. Therefore, even the most experienced professionals could occasionally make mistakes, as visual identification and verification are prone to human error, especially in high-pressure and high-volume environments such as hospitals and clinics [7]. These challenges and limitations have led to investments in automation technologies for unit-dose dispensing to improve hospital pharmacy services [8,9] and the exploration of leveraging novel techniques, such as object detection, to assist pharmacists with verification tasks and ultimately reduce dispensing errors [6]. However, it is important to note the specific clinical context for such tools. Unlike medication reconciliation at admission, where a patient may present a disorganized collection of loose pills for identification, during hospital pharmacy manual dispensing processes, staff typically use organized unit doses and dosette boxes. In this context, the primary challenge is usually not identifying a mystery pill but verifying that the pill in a specific compartment matches the prescribed therapy. Automated pill recognition systems could support this verification process, as well as additional workflows, including detection of dispensing errors before medications reach patients and identification of medications returned to the hospital pharmacy unit.

Recent advances in computer vision and object detection offer promising solutions for image analysis, including high-performance object detection models based on the You Only Look Once (YOLO) architecture, which allows fast and accurate localization and classification in a single step [10,11]. YOLO has proven to be an attractive choice for a variety of medical applications [11], including pill recognition [12]. YOLO has demonstrated strong performance in code-based pill recognition systems, particularly for preprocessing segmented images within multistream, 2-phase neural models using metric embedding [13]. One of the latest iterations in the Ultralytics YOLO series is YOLO11, released in September 2024. This version improved upon previous models by implementing a new backbone architecture that enhances feature extraction (identifying key patterns such as edges, textures, or shapes from raw image data) with improved accuracy and processing speed, making it suitable for complex visual recognition tasks [14]. These advancements create new opportunities for developing clinical decision support systems that assist health care professionals in the visual identification of pills.

In parallel with the evolution of code-based models, which provide flexibility and customization but require strong programming skills and longer development time, low-code or code-free automated machine learning (AutoML) platforms have emerged. These platforms enable relatively simple development and testing of specialized object detection models using easy-to-use cloud-based platforms [15,16].

### Rationale

Although both traditional code-based models and cloud-based AutoML platforms have shown promise in medical image analysis [17], there is a lack of comprehensive, direct comparative studies evaluating the performance of these approaches in the context of pill recognition. Previous studies have commonly focused on controlled environments that do not reflect typical usage scenarios and conditions. The open-source, code-based deep learning object detection YOLO model and code-free, cloud-based AutoML platforms represent fundamentally different development concepts, each with its own associated advantages and limitations. This justifies a comprehensive comparison of these solutions to determine robustness, scalability, and implementation complexity in practical environments. Limited published, peer-reviewed studies compare the performance of traditional code-based and code-free pill recognition models using images captured in real-world clinical environments, which often differ substantially from standardized reference images. To our knowledge, this is the first study to directly compare these approaches using clinical data under such specific conditions.

### Objectives

This study aimed to evaluate and compare the effectiveness of pill recognition models developed using YOLO11 with cloud-based AutoML platforms provided by Amazon Rekognition Custom Labels, Google Vertex artificial intelligence (AI) AutoML Vision, and Microsoft Azure Custom Vision. Factors, such as cost, ease of use, and deployment feasibility, as well as key performance metrics including accuracy, precision, recall, *F*_1_-score, mean average precision (mAP), overall error rates (OERs), and false negative rates (FNRs), were evaluated on different test sets, including real-world images captured by clinical pharmacists.

### Code-Based and Code-Free Object Detection

Traditional code-based deep learning frameworks, such as YOLO11, require programming and machine learning (ML) expertise but offer full control over model architecture and training parameters, whereas code-free development and training using cloud-based AutoML platforms provide simplified workflows with limited customization options. The YOLO family of deep-learning–based object detectors uses a single-stage approach, which means that the entire input image is processed in a single forward pass, predicting bounding boxes and class probabilities simultaneously [18]. This allows YOLO models to balance inference speed and detection accuracy, making them one of the most widely used object detection algorithms. In our experiments, we used the YOLO11 real-time object detection model, which was designed to address a wide spectrum of application requirements and supports a wide range of computer vision tasks, including object detection [14,18]. A detailed description of the YOLO11 model structure is provided in Multimedia Appendix 1.

**Figure 1.**
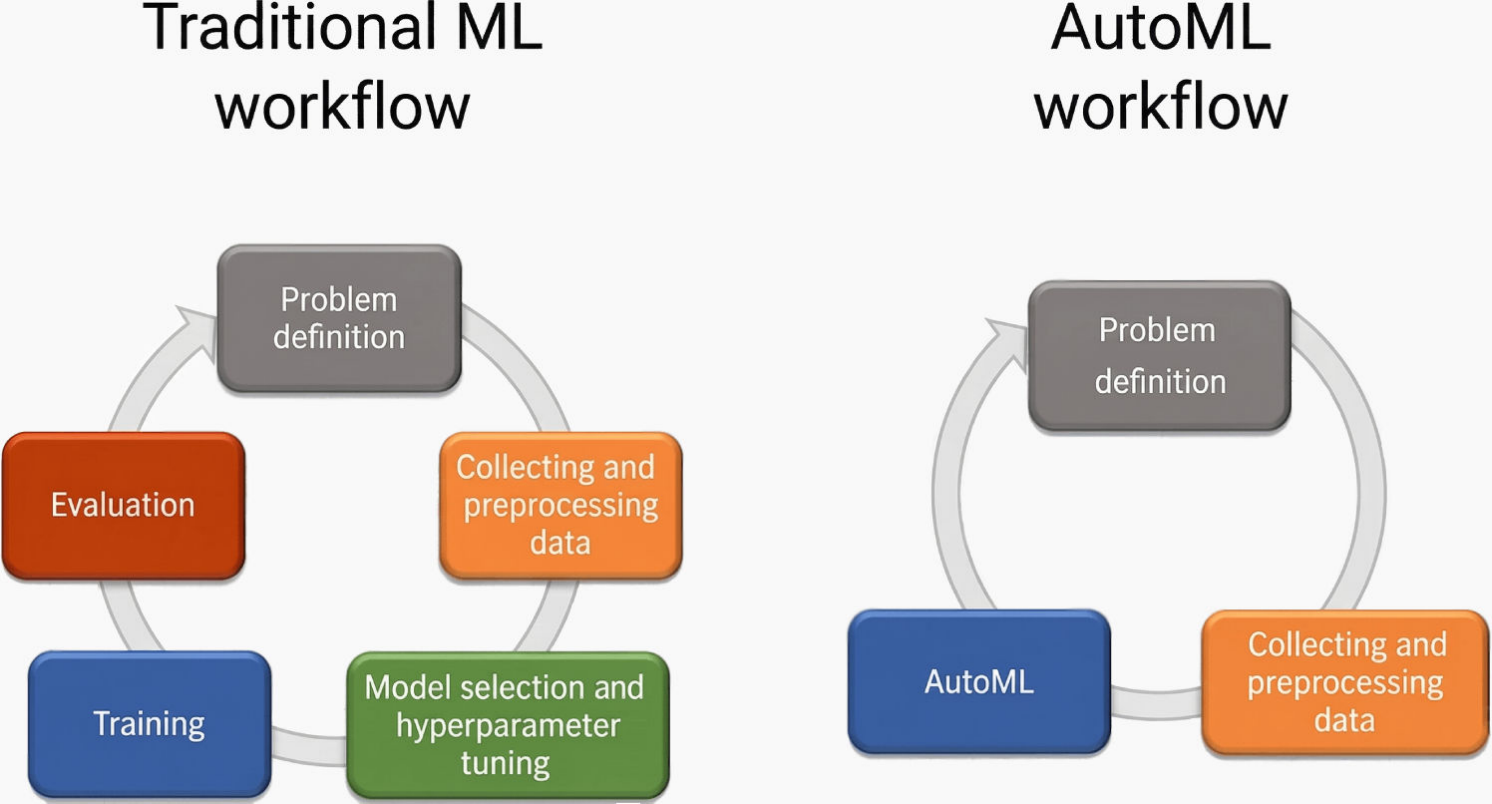
Traditional machine learning (ML) vs automated machine learning (AutoML) workflows.

In a traditional deep learning ecosystem, model development involves several manual and iterative processes, such as data collection and preprocessing, feature engineering, model selection, hyperparameter tuning, training, and validation, as shown in Figure 1. This approach requires a high level of expertise and intensive experimentation to fine-tune the model’s performance.

Cloud-based, no-code AutoML platforms have emerged as suitable alternatives to traditional, often code-intensive model development approaches. These platforms automate most of the workflows involved in managing ML pipelines, thereby lowering the barrier to entry and making model development accessible even to those without programming skills or data science expertise [19]. In addition to enhancing accessibility, these platforms accelerate the model development lifecycle by automating repetitive and time-consuming tasks, allowing rapid prototyping, while reducing complexity by handling the underlying tasks associated with managing the computational infrastructure and ecosystem required for model development [19]. However, AutoML has inherent disadvantages. While AutoML democratizes model development by minimizing human effort, it requires significant computational resources, which can lead to expensive cloud service costs, particularly when developing models iteratively using large datasets. AutoML users typically have very limited control over the choice of base models, underlying training algorithms, and hyperparameter adjustments. In addition, AutoML platforms lack transparency in model architecture and hyperparameters and may raise privacy concerns [20]. Therefore, the choice of the optimal platform depends on a variety of factors.

This study aimed to provide empirical evidence to guide such platform selection decisions for pill recognition model development intended for deployment in clinical and hospital pharmacy settings, where accuracy and reliability are of utmost importance. We evaluated AutoML solutions offered by Amazon Web Services (AWS), Google Cloud, and Microsoft Azure, 3 of the most popular cloud-based providers for object detection, alongside code-based models trained with a traditional workflow using YOLO11.

Microsoft offers a wide range of AI and ML services on its Azure platform, including Custom Vision, a more specialized service focused on building, training, and deploying custom image classification and object detection models using a no-code or low-code approach [21]. It is accessible through a dedicated, code-free web interface and software development kits (SDKs) requiring minimal coding experience. Vertex AI AutoML Vision is part of Google Cloud’s unified Vertex AI platform. Vertex AI allows users to train, deploy, and fine-tune a wide range of models with supervised learning, data- and application-specific training strategies, and tools to manage the entire lifecycle of ML [22]. Vertex AI offers choices for a manual approach that provides full control over the training pipeline using traditional coding development, as well as AutoML for code-free model development, which is accessible via the Google Cloud Console web interface and through SDKs compatible with popular open-source libraries such as TensorFlow (Google) and PyTorch (Meta AI) [23]. Amazon Rekognition Custom Labels is a feature within AWS’s broader Rekognition service that allows users to train custom object detection models using AutoML [24]. Although Amazon Rekognition follows a similar workflow to other platforms, including the option of manual labeling, it imposes stricter restrictions on the ML workflow.

## Methods

### Overview

This section details the methodologies used for data collection, preprocessing, and coded model development. We compared 2 fundamentally different approaches to model development and training: traditional code-based deep learning using YOLO11, and code-free development using 3 major cloud-based AutoML platforms (Amazon Rekognition Custom Labels, Vertex AI AutoML Vision, and Custom Vision). We performed 5 training runs per platform using 5 progressively larger subsets of our training images. The evaluation metrics used for benchmarking and comparative analysis are explained in detail in Multimedia Appendix 2.

### Data Collection and Preprocessing

**Figure 2.**
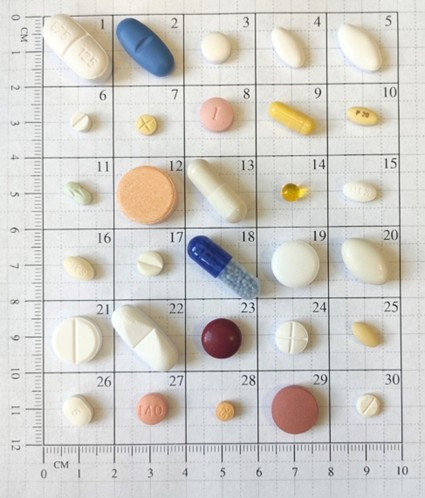
The 30 medications selected for this study*.*

For model training and evaluation, we used a tailor-made proprietary dataset focusing on 30 commonly dispensed solid oral dosage forms in Hungary (Figure 2). Selection of medications and curation of the dataset followed a strict protocol described in our previous publication [15].

**Figure 3.**
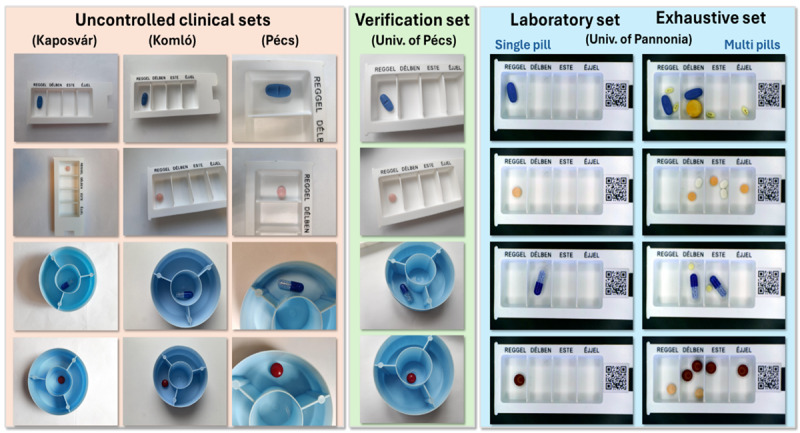
Illustration of testing datasets*.*

For testing datasets, images were captured with a single pill placed in blue-and-white dosette boxes routinely used for manual dispensing in Hungarian clinical settings, except for one dataset prepared for exhaustive performance evaluation, which contained multiple pills per image (refer to Figure 3).

Training images had a resolution of 1200×900 pixels, while test images were at native device resolution (4032×3024 and 4000×3000 pixels) to better simulate real-world conditions where input images vary in size. All images were captured in JPEG format with consistent quality settings.

The study’s datasets were organized as follows:

- The “full training dataset” comprised 26,880 images in total, provided by the University of Pécs, with 896 images per class for the 30 selected medications. To evaluate the impact of training data size on the model performance, 5 subsets were created from these images using a logarithmic binning strategy, each with a progressively increasing number of images:
  - Subset 1: 1230 images (41 images/class)
  - Subset 2: 3450 images (115 images/class)
  - Subset 3: 7380 images (246 images/class)
  - Subset 4: 14,400 images (480 images/class)
  - Subset 5: 26,880 images (full training dataset, 896 images/class)
- A “verification dataset*”* containing 1440 images (48/class) was created at the University of Pécs using the same location, conditions, and procedures as the training dataset images.
- Three *“*uncontrolled clinical test sets (UCTSs)” of 480 images each (16/class) were captured by clinical pharmacists at Kaposvár, Komló, and Pécs hospitals under authentic clinical conditions to assess model performance in uncontrolled health care environments. UCTS images were collected using purposive sampling to ensure balanced representation across all medication classes (16 images/class at each hospital site). This design enables fair per-class comparison of model performance, avoiding bias toward majority classes seen with imbalanced test sets. Similar balanced sampling approaches are used in established pill recognition benchmarks, such as the CURE dataset [25] and the National Library of Medicine Pill Image Recognition Challenge [26]. To create the dataset, clinical pharmacists photographed medications within their routine workspaces using the dosette boxes and imaging equipment available in their daily practice. Class identity recorded during image acquisition served as the ground truth label during subsequent evaluations. This protocol preserved authentic environmental and human variables affecting input image quality, including camera variability, lighting conditions, and operator technique, while maintaining balanced class representation for fair platform comparison. No image quality filtering or manipulation was applied; all captured images were included regardless of quality to ensure the dataset accurately reflected authentic real-world variability in clinical settings.
- Two additional test datasets were created in a controlled laboratory environment at the Image Processing Laboratory, University of Pannonia. The first dataset, referred to as “laboratory test set,*”* contained 120 images with single pills per image, while the second dataset included 120 multipill images called “exhaustive test set” intended for exhaustive model testing. Medications were arranged in a white dosette box, and controllable top-mounted lighting was used to evenly illuminate the pills. Images were captured with a fixed camera position at 3840×2160 resolution, with a slight reduction in resolution following image undistortion.

### YOLO11 Implementation

For our experiments, we selected the medium-sized pretrained model (YOLO11-m), which balances speed, accuracy, and training time. All hyperparameters remained at their default values, except for the early stopping patience parameter, which was set to 15 epochs to terminate training if the validation loss did not improve within 15 consecutive epochs. Standard data augmentation techniques (eg, rotation, brightness adjustment, and so on) were not applied during training to ensure uniform conditions across platforms, as cloud-based AutoML platforms typically apply such augmentations automatically, with limited or no user control. All experiments ran for 100 epochs with a batch size of 20, using an NVIDIA Quadro RTX 5000 graphics processing unit (GPU) with 16 GB video random-access memory for both training and evaluation.

### Cloud-Based AutoML Platform Implementation

#### Microsoft Azure Custom Vision

Custom Vision offers a streamlined workflow for training object detection models. Users must create Custom Vision training and prediction resources in their Azure subscription via the Azure portal. New object detection projects can be created through the Custom Vision portal or via SDK, which offers a choice of predefined domains optimized for specific scenarios and edge or mobile deployment. Training images can be uploaded directly via the web portal or SDK, with an integrated tagging interface for drawing bounding boxes and assigning labels. Custom Vision requires a minimum of 15 images per label for training, but recommends 50 or more images per label for better performance. Users can select either Quick training (completed in minutes) or Advanced training (with extended computation budget) [27]. The platform automatically selects the optimal base model, training, tuning, and augmentation settings based on uploaded data and the selected project domain. Each training run generates a new model iteration that can be published to a prediction endpoint accessible via Representational State Transfer application programming interfaces (APIs) or SDKs.

#### Google Vertex AI AutoML

Vertex AI requires an active Google Cloud project with images stored in Google Cloud Storage. The platform accepts various image formats, with data imported either from local machines or directly from Google Cloud. Annotations must be provided in comma-separated values or JSON Lines (JSONL) formats containing bounding box coordinates and corresponding labels. Vertex AI requires at least 10 annotated images per label for training and recommends providing 1000 or more for optimal model performance [28]. For unannotated datasets, users can apply annotations through the Google Cloud Console interface. Without user-specified splitting, Vertex AI automatically partitions datasets using an 80%‐10%‐10% split for training, validation, and testing [29]. Since our original XML annotations were incompatible with Vertex AI’s input requirements, we converted them to a single JSONL file, with each line representing an annotated instance. We created the dataset by selecting object detection as the project objective and linking the JSONL file from a cloud storage bucket. Vertex AI’s custom dataset splitting functionality was used to create appropriate training, validation, and testing sets. For optimization, we used the high-accuracy training option within Vertex AI. After performing initial experiments, we opted for a specific high-accuracy training configuration that significantly outperformed other available options.

#### Amazon Rekognition Custom Labels

Rekognition requires prelabeled data in Amazon SageMaker Ground Truth manifest format, a JSONL structure with pixel-based bounding box coordinates [30]. This format has a different internal structure compared to Vertex AI’s requirements, necessitating the conversion of our annotations using Python scripts. Data management in Rekognition offers moderate flexibility; however, Amazon S3 is the mandatory cloud solution for image storage. If no test dataset is provided, Rekognition automatically splits the training data to create a test dataset using an 80%‐20% split [31].

Model training can be initiated through the AWS Console or the CreateProjectVersion API. However, Rekognition provides no hyperparameter control, limiting customization during training. Each successful training run generates a new project version with an associated Amazon Resource Name. Performance metrics, including *F*_1_-score, precision, recall, and mAP, are available through the API and Rekognition.

### Evaluation Metrics

Object detection evaluation differs from standard classification because it requires assessment of localization and classification, meaning models must simultaneously locate objects within an image and correctly identify each object. Therefore, metrics that ignore spatial accuracy of predictions, such as simple classification accuracy, precision, and recall without intersection over union (IoU) thresholds, or classification-only *F*_1_-scores, are insufficient for rigorous model evaluation.

To comprehensively assess our pill recognition models for both localization precision and classification accuracy, we used metrics incorporating IoU to enable fair comparison across fundamentally different model architectures and development approaches.

IoU quantifies the spatial accuracy of object detection by measuring the overlap between predicted and ground-truth bounding boxes, calculated as the ratio of overlap area to union area between boxes. Higher values indicate better localization accuracy, with a threshold (typically 0.5) determining whether a detection is considered correct.

In object detection, the accuracy metric is adapted to account for both classification and localization performance. A prediction is considered correct only if it has the correct class label and the IoU with the ground-truth bounding box exceeds a specified threshold.

Precision and recall incorporate IoU thresholds for object detection. Precision measures the percentage of correct detections among all predictions, indicating how reliable the model’s positive predictions are. Recall measures the percentage of ground-truth objects correctly detected, reflecting the model’s ability to identify all relevant instances.

Average precision represents the area under the precision-recall curve, providing a comprehensive performance measure across different confidence thresholds for each class. mAP averages average precision values across all classes, providing an overall performance measure. Common variants include mAP@0.50 (using an IoU threshold of 0.5) and mAP@0.50‐0.95 (averaging across multiple IoU thresholds from 0.5 to 0.95 in 0.05 increments), which provides a more rigorous evaluation.

Beyond standard performance metrics, a critical evaluation of model suitability for clinical deployment necessitates an analysis of error profiles. To understand the failures impacting patient safety, we analyzed OERs and FNRs for each model. OER and accuracy are inversely related; they measure the model’s overall tendency to misidentify medications through missed detections or incorrect classifications. FNR specifically quantifies the proportion of pills that models fail to detect, a critical safety metric in clinical settings. A detailed description of evaluation metrics, mathematical definitions, and computational procedures is provided in Multimedia Appendix 2.

### Analytical Approach

This study used a comparative benchmarking design to characterize platform performance across diverse deployment scenarios rather than hypothesis testing. We selected standard object detection metrics following established conventions in computer vision and pill recognition research to enable direct comparison with other published literature. We additionally reported OER and FNR due to their clinical relevance for patient safety. CIs and formal statistical comparisons across hospital sites were not performed, as the evaluated cloud-based AutoML platforms have major inherent limitations and do not consistently provide the data needed for these calculations.

### Used Medicines

Our datasets included the 30 most commonly dispensed medications from 3 participating clinical centers [15]. The medications represent various therapeutic areas, including cardiovascular agents (eg, bisoprolol and perindopril), antibiotics (amoxicillin/clavulanic acid), analgesics (naproxen and tramadol), psychiatric medications (alprazolam and quetiapine), and essential vitamins and supplements.

These medications cover a diverse range of solid oral dosage forms, including film-coated and uncoated tablets (the most prevalent forms), enteric-coated tablets, hard and soft gelatin capsules, and one chewable tablet. A complete medication list with active pharmaceutical ingredients, dosage forms, distinctive features, colors, and shapes is provided in Multimedia Appendix 3.

### Ethical Considerations

This study did not involve human participants, and ethics approval was therefore not required. No personally identifiable information was collected or processed. No informed consent was required, as no human participants were involved in this study.

## Results

### Overall Detection Performance

Our evaluation of pill recognition models across multiple test datasets revealed notable performance differences, with no single platform consistently dominating all test scenarios. We trained models using 5 progressively larger training subsets (from 1230 to 26,880 images) and evaluated them using standard metrics: accuracy, precision, recall, OER, FNR, *F*_1_-score, mAP@0.50, and mAP@0.50‐0.95 to comprehensively assess platform performance, strengths, and limitations. Table 1 presents the results of models trained on the full dataset of 26,880 images, with comprehensive results of all training runs and subsequent evaluations provided in Multimedia Appendix 4.

**Table 1.** Summary of platform performance across all testing datasets (trained with the full training dataset of 26,880 images).

Platform and dataset	Accuracy (%)	Precision	Recall	OER^a^	FNR^b^	*F*_1_-score	mAP@0^c^.50	mAP@0.50‐0.95
Ultralytics YOLO11								
Kaposvár	80.63	0.98	0.92	0.19	0.08	0.95	0.99	0.62
Komló	78.96	1.00	0.91	0.21	0.09	0.95	1.00	0.72
Pécs	64.58	0.99	0.91	0.35	0.09	0.95	0.99	0.76
Verification	80.83	1.00	0.96	0.19	0.04	0.98	1.00	0.89
Laboratory	40.00	1.00	0.87	0.60	0.13	0.93	0.96	0.48
Exhaustive	44.10	0.99	0.81	0.56	0.19	0.89	0.95	0.40
Google Vertex AI^d^								
Kaposvár	71.04	0.96	0.90	0.29	0.10	0.93	0.97	0.53
Komló	90.00	1.00	0.96	0.10	0.04	0.98	1.00	0.58
Pécs	65.42	1.00	0.81	0.35	0.19	0.90	0.97	0.67
Verification	91.60	1.00	0.98	0.08	0.02	0.99	1.00	0.79
Laboratory	67.50	1.00	0.86	0.33	0.14	0.92	1.00	0.54
Exhaustive	52.79	1.00	0.66	0.47	0.34	0.79	0.99	0.45
Microsoft Azure Custom Vision								
Kaposvár	62.71	0.98	0.80	0.37	0.20	0.88	0.99	0.58
Komló	77.71	0.99	0.92	0.22	0.08	0.95	1.00	0.67
Pécs	56.04	0.98	0.81	0.44	0.19	0.89	0.99	0.65
Verification	85.62	1.00	0.94	0.14	0.06	0.97	1.00	0.70
Laboratory	51.67	0.97	0.78	0.48	0.22	0.86	0.98	0.59
Exhaustive	33.61	0.92	0.54	0.66	0.46	0.68	0.77	0.33
Amazon Rekognition								
Kaposvár	70.63	1.00	0.76	0.29	0.24	0.86	0.76	0.45
Komló	77.71	1.00	0.80	0.22	0.20	0.89	0.79	0.53
Pécs	20.62	0.92	0.26	0.79	0.74	0.40	0.26	0.19
Verification	84.72	1.00	0.85	0.15	0.15	0.92	0.85	0.71
Laboratory	43.33	1.00	0.80	0.57	0.20	0.89	1.00	0.77
Exhaustive	41.97	0.99	0.76	0.58	0.24	0.86	0.98	0.67

a OER: overall error rate.

b FNR: false negative rate.

c mAP: mean average precision.

d AI: artificial intelligence.

Results are organized into 3 categories: UCTS (representing real-world hospital conditions), verification dataset (optimal imaging conditions replicating training images), and laboratory-controlled datasets (standardized research settings).

On the UCTS images from Kaposvár hospital, models trained with the full training dataset of 26,880 images exhibited varied performance (Table 1). YOLO11 demonstrated superiority, achieving the highest scores in accuracy (80.63%), recall (0.92), *F*_1_-score (0.95), and mAP metrics. Amazon Rekognition achieved the highest precision (1) but also showed the highest FNR, indicating its conservative detection strategy. Vertex AI ranked second with 71.04% accuracy. Microsoft Azure did not achieve top results in any instance on this dataset, but it maintained consistent performance, with accuracy ranging from 56.87% to 62.92% across training set sizes (Multimedia Appendix 4).

On the UCTS images from Komló hospital, competition was balanced between YOLO11, Custom Vision, and Amazon Rekognition, while Vertex AI showed considerably higher performance compared to the others. Across all training configurations, YOLO11 dominated mAP metrics, achieving a perfect 1.0 mAP@0.50 with 14,400 and 26,880 training images. Vertex AI excelled in accuracy, reaching 90.62% with 7380 images and 90% with the full training subset of 26,880 images. Custom Vision improved steadily from 69.58% to 77.71% accuracy with increasing training data, while Amazon Rekognition maintained perfect precision (1.0) across all 5 training scenarios while achieving 77.71% accuracy.

On the UCTS images from Pécs hospital, Vertex AI outperformed other platforms despite varying results, achieving 79.79% accuracy with the training subset of 14,400 images but 65.42% with the full dataset. The other AutoML solutions performed poorly: Microsoft Azure achieved 57.5% accuracy with the training subset of 14,400 images and 56.04% with the full training dataset, while Amazon Rekognition reached only as high as 27.08% accuracy with the training subset of 7380 images on this dataset, which fell to 20.62% when trained with the full training dataset (Multimedia Appendix 4). YOLO11 also showed strong results, particularly for the more challenging mAP@0.50‐0.95 threshold metric (0.76). This dataset demonstrated the challenging nature of certain clinical environments, where lighting conditions, camera positioning, and other external factors significantly impact model performance.

On the verification dataset from the University of Pécs, Vertex AI excelled across all metrics, achieving 91.60% accuracy, perfect 1.0 precision and mAP@0.50, and 0.98 recall with the full training dataset. It only slightly underperformed YOLO11 in the mAP@0.50‐0.95 metric (0.79 vs 0.89). Microsoft Azure achieved 85.62% accuracy, 1.0 precision, and 1.0 mAP@0.50, while Amazon Rekognition reached 84.72% accuracy with perfect precision (1.0) but lower recall (0.85). YOLO11 showed linear performance improvement with increasing training dataset size, reaching 80.83% accuracy with perfect precision (1.0) and a high recall of 0.96. This dataset demonstrated the potential upper bound on performance when testing conditions are well-matched to training data.

**Figure 4.**
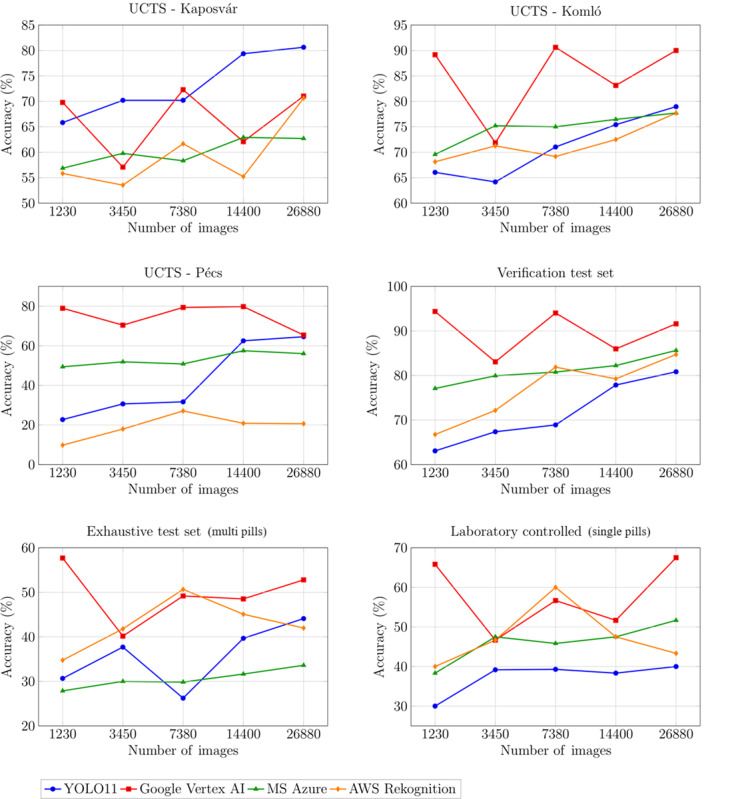
Accuracy of object detection models across datasets. AI: artificial intelligence; AWS: Amazon Web Services; UCTS: uncontrolled clinical test set.

The laboratory-controlled (single pills) and the exhaustive (multipills) test sets from the University of Pannonia showed varying performance across different platforms under optimal imaging conditions in a fully controlled environment. When analyzing the exhaustive dataset, Vertex AI achieved the highest accuracy on the full training dataset (52.79%) with excellent mAP@0.50 (0.99), followed by YOLO11 (44.10%, 0.95), Amazon Rekognition (41.97%, 0.98), and Microsoft Azure (33.61%, 0.77). The lower accuracy scores in the exhaustive dataset reflect the increased complexity of detecting multiple objects within an image, with potential occlusions and varying orientations. On the laboratory dataset, Vertex AI achieved 67.50% accuracy with perfect 1.0 precision and mAP@0.50, followed by Amazon Rekognition, mainly due to its robust mAP metrics, which maintained good performance across other metrics, although still falling short of Vertex AI’s performance. Figure 4 visualizes these trends across all datasets and training set sizes, illustrating nonlinear performance patterns and platform-specific variations.

### Influence of Training Dataset Size on Model Performance

Increasing training images generally improved model performance across all metrics, although improvements were nonlinear with platform-specific patterns. Table 2 illustrates these effects using the verification dataset, which, despite being captured under optimal conditions similar to training data, serves as a neutral baseline for comparing platform responses to increased training data because all models were trained and tested using the same datasets. Overall, YOLO11 and Custom Vision showed predictable linear improvement, with YOLO11’s accuracy increasing from 63.06% to 80.83% and Microsoft Azure’s from 77.08% to 85.62% as training data increased from 1230 to 26,880 images. Conversely, Amazon Rekognition and Vertex AI showed variable patterns with several fluctuations, suggesting dynamic model selection based on dataset characteristics, resulting in performance variations that do not follow the expected logarithmic improvement pattern with an eventual plateau typically seen in ML models. Notably, Vertex AI achieved near-optimal performance (94.37% accuracy, 1.0 precision, and 1.0 recall) with the smallest training subset at just 1230 images, though challenging clinical datasets required the full training set for acceptable performance.

**Table 2.** Effect of training dataset size on model performance on the verification dataset.

Platform and training subset	Accuracy	Precision	Recall	OER^a^	FNR^b^	*F*_1_-score	mAP@0^c^.50
Ultralytics YOLO11							
1230 images	63.06%	1.00	0.85	0.37	0.15	0.92	1.00
3450 images	67.36%	1.00	0.86	0.33	0.14	0.93	1.00
7380 images	68.89%	1.00	0.91	0.31	0.09	0.95	1.00
14,400 images	77.85%	1.00	0.96	0.22	0.04	0.98	0.95
26,880 images	80.83%	1.00	0.96	0.19	0.04	0.98	1.00
Google Vertex AI^d^							
1230 images	94.37%	1.00	1.00	0.06	0.00	1.00	1.00
3450 images	83.06%	1.00	0.98	0.17	0.02	0.99	1.00
7380 images	94.03%	1.00	0.99	0.06	0.01	0.99	1.00
14,400 images	85.97%	1.00	0.98	0.14	0.02	0.99	1.00
26,880 images	91.60%	1.00	0.98	0.08	0.02	0.99	1.00
Microsoft Azure Custom Vision							
1230 images	77.08%	1.00	0.91	0.23	0.09	0.95	1.00
3450 images	79.93%	1.00	0.92	0.20	0.08	0.96	1.00
7380 images	80.76%	1.00	0.90	0.19	0.10	0.95	1.00
14,400 images	82.22%	1.00	0.93	0.18	0.07	0.96	1.00
26,880 images	85.62%	1.00	0.94	0.14	0.06	0.97	1.00
Amazon Rekognition							
1230 images	66.74%	1.00	0.68	0.33	0.32	0.81	0.72
3450 images	72.15%	1.00	0.75	0.28	0.25	0.86	0.79
7380 images	81.87%	1.00	0.84	0.18	0.16	0.91	0.84
14,400 images	79.24%	1.00	0.81	0.21	0.19	0.89	0.82
26,880 images	84.72%	1.00	0.85	0.15	0.15	0.92	0.85

a OER: overall error rate.

b FNR: false negative rate.

c mAP: mean average precision.

d AI: artificial intelligence.

### Cost, Usability, and Limitations of AutoML Platforms

Several important pricing and usability differences exist between platforms. Vertex AI and Amazon Rekognition Custom Labels charge continuously for endpoint availability, accumulating costs in minute increments even during idle periods. In contrast, Custom Vision charges only per request, eliminating costs during idle periods. Vertex AI maintains consistent flat-rate pricing regardless of the dataset size, charging US $3.465 per hour for training and US $2.002 per hour for deployment and online prediction [32]. Amazon Rekognition Custom Labels charges US $1 per hour for training and US $4 per hour for inference, but scales training time and resources with dataset size, resulting in proportionally higher costs for larger datasets [33]. Custom Vision charges US $10 per hour for training, but allows users to specify computation time budgets, providing better cost control. Inference costs US $2 per 1000 individual requests [34]. While Google and Amazon have regional pricing variations, Azure maintains uniform US dollar pricing that is directly converted to local currencies. Each platform offers limited free tiers: Azure provides 1 hour of free training per month plus 10,000 predictions; Google provides US $300 in free credit for new customers and includes 30 minutes of free online predictions; Amazon offers 1 free inference hour per month during its 12-month free tier. YOLO11, being open-source, has no platform costs but requires local computational resources.

Training times varied considerably. YOLO11 training times scaled linearly with dataset size, from 38 minutes for the 1230-image training subset to nearly 13 hours for the full dataset. Microsoft Azure completed all training within 3 hours, even when allocated 4‐5 hours, indicating that the cloud-based AutoML platform determined that additional training was unnecessary. Amazon Rekognition required the longest time, reaching nearly 40 hours for the full dataset. Vertex AI maintained consistent 2.5‐3-hour training times regardless of dataset size. Table 3 provides an overview of training times and associated costs for each platform.

**Table 3.** Training time and cost comparison across platforms.

Platform and training subset	Training time (hour:minute)	Training cost (US $)
Ultralytics YOLO11		
1230 images	00:38	<1
3450 images	01:41	<1
7380 images	03:37	<1
14,400 images	06:53	<1
26,880 images	12:53	<1
Google Vertex AI^a^		
1230 images	02:38	69.30
3450 images	02:48	69.30
7380 images	02:53	69.30
14,400 images	02:58	69.30
26,880 images	02:59	69.30
Microsoft Azure Custom Vision		
1230 images	00:57	9.50
3450 images	01:03	10.50
7380 images	01:02	10.33
14,400 images	01:37	16.10
26,880 images	02:52	28.60
Amazon Rekognition		
1230 images	04:55	5.43
3450 images	09:28	10.48
7380 images	17:04	18.88
14,400 images	25:18	27.98
26,880 images	39:41	43.89

a AI: artificial intelligence.

### Performance on Edge Device

We evaluated Microsoft Azure and YOLO11 on a Raspberry Pi 5 using the exhaustive test set. Only these platforms were tested since Microsoft Azure alone provided downloadable Open Neural Network Exchange (ONNX) models for edge deployment. Table 4 shows a significant performance trade-off between edge and cloud-based inference.

Microsoft Azure’s performance decreased on-device (25.83% accuracy) compared to cloud inference (33.61% accuracy). YOLO11 maintained better consistency, achieving 43.33% accuracy with ONNX and 44.10% with the native .pt format. Inference times differed substantially: Microsoft Azure ONNX achieved 0.19 seconds per prediction, while YOLO11 required 1.38 seconds per prediction in ONNX format on the Raspberry Pi 5.

**Table 4.** Performance metrics comparison for edge deployment.

Platform and weight format^a^	Accuracy	Precision	Recall	*F*_1_-score	mAP^b^ @0.50	mAP@0.50‐0.95	Average prediction time (seconds)
Ultralytics YOLO11							
ONNX^c^	43.33%	1.00	0.85	0.92	0.93	0.44	1.38
PyTorch (Meta AI; .pt)	44.10%	0.99	0.81	0.89	0.95	0.34	0.08
Microsoft Azure							
ONNX	25.83%	0.84	0.57	0.68	0.74	0.29	0.19
API^d^ call	33.61%	0.92	0.54	0.68	0.77	0.33	0.83

a Models trained with the full dataset of 26,880 training images; metrics calculated at a confidence level of 0.5 and an intersection over union (IoU) threshold of 0.5.

b mAP: mean average precision.

c ONNX: Open Neural Network Exchange.

d API: application programming interface.

## Discussion

### Principal Findings

Our comprehensive evaluation of pill recognition models using code-based YOLO11 vs major code-free cloud-based AutoML platforms revealed that no single platform consistently outperforms others across all test scenarios, highlighting the importance of matching platform selection to specific deployment conditions and available training resources. The dramatic performance variations observed across clinical sites—for example, ranging from 20.62% accuracy (Amazon Rekognition) to 65.42% accuracy (Vertex AI) on the same clinical hospital dataset when both were trained on the full 26,880-image training dataset—demonstrate that imaging hardware, environmental, and personnel factors have a more tangible impact on model performance than platform choice alone.

Our results indicate that increasing the number of training images generally improves model performance across all metrics, although improvement patterns were not strictly linear and varied by platform, likely due to different underlying architectures and optimization strategies. Fair comparisons are challenging, especially for AutoML frameworks, which dynamically select the most suitable model architecture for each specific dataset and task. Consequently, different model types may be chosen (eg, a convolutional neural network in one instance and a vision transformer in another), even when trained on the same dataset.

Another critical finding is the substantial variation in how platforms balance the precision-recall trade-off across real-world clinical datasets. Amazon Rekognition’s high precision across multiple clinical datasets often came at the cost of low recall, with values as low as 0.26 in one clinical dataset. This conservative approach resulted in the platform missing up to 74% of pills in the most challenging clinical dataset. Conversely, YOLO11’s balanced precision-recall trade-off suggests suitability for scenarios where both false positives and false negatives carry clinical risk.

YOLO11 demonstrated the most consistent and predictable improvement trajectory with larger training datasets, achieving excellent mAP scores on most datasets, making it ideal for applications requiring precise object localization. The most dramatic improvement was observed on the most challenging clinical dataset, where accuracy increased from 22.71% to 64.58% and recall improved from 0.49 to 0.91. Consistently positive mAP trends across all datasets confirmed YOLO11’s ability to effectively use additional training data.

Vertex AI presented more complex patterns. While generally improving with larger training datasets, performance fluctuations, including degradations, occurred in multiple instances with larger subsets. For instance, on the same clinical dataset, its accuracy dropped from 69.79% (1230-image training subset) to 57.08% (3450-image training subset), then recovered to 72.29% (7380-image training subset), dropped again to 62.08% (14,400-image training subset), and finally reached 71.04% (26,880-image full training dataset). This pattern likely reflects dynamic model architecture selection, where different model types are chosen for different dataset sizes. While resource-efficient, this behavior introduces unpredictability that could complicate training and deployment.

Custom Vision showed the most consistent behavior among cloud-based AutoML platforms, maintaining stable performance without degradation risks from model changes. Although Microsoft does not publicly disclose the model architecture used by Custom Vision, analyses of exported ONNX models suggest that the structure appears to be an older YOLOv2-based architecture, which would explain this consistency and the absence of architectural uncertainty we observed in other cloud-based AutoML platforms. Performance improvement trends paralleled YOLO11; however, the older architecture resulted in lower overall metrics.

**Figure 5.**
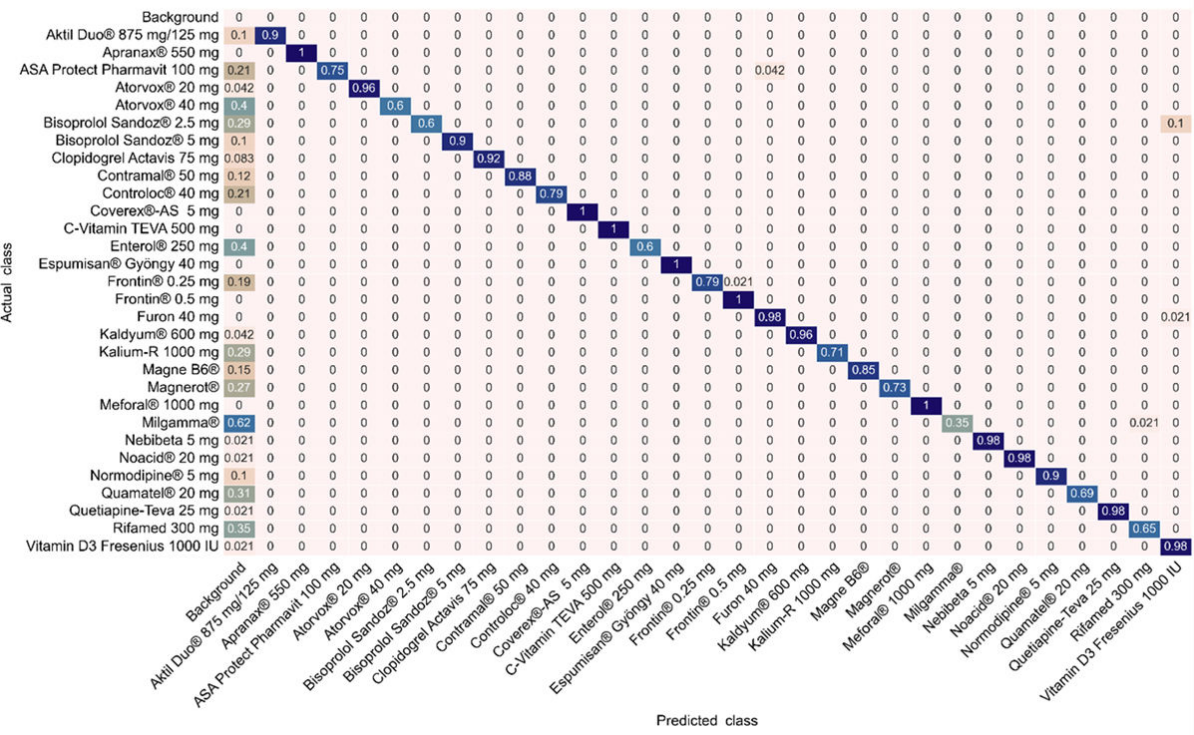
Confusion matrix showing Amazon Rekognition performance on the verification dataset (trained on the full training dataset of 26,880 images).

Amazon Rekognition improved consistently with training data volume despite performing dynamic model selection during training. It demonstrated particularly strong performance on fully controlled datasets, especially in mAP values, with remarkable stability across IoU thresholds. Unlike other platforms, it showed minimal degradation at the 0.50 IoU threshold and maintained strong performance on the challenging mAP@0.50‐0.95 metric. It follows a conservative object detection strategy that prioritizes avoiding false positives, maintaining perfect precision (1.0) across multiple tests but with considerable false negative values indicative of missed detections, illustrated in the confusion matrix (Figure 5) with values listed in the background column. Analysis of confusion patterns revealed that the primary source of misclassification across all platforms was visual similarity among medications. Small, round, oblong, and oval white or off-white pills were most frequently confused with each other. Notably, these visually similar medications often represent therapeutically distinct drug classes (eg, beta-blockers, antiplatelet agents, and anxiolytics), where misidentification could have significant clinical consequences. Medications with distinctive visual characteristics, such as unique colors, shapes, or prominent surface markings, showed substantially lower misclassification rates across all platforms. The confusion matrices in Figure 5 and Multimedia Appendix 5 illustrate these visual and platform-specific confusion patterns in detail.

The cost-performance evaluation showed that although cloud-based AutoML platforms offer high accessibility, they also incur high platform costs, whereas traditional approaches, such as YOLO11, may provide comparable or better performance without platform costs. However, this does not account for the technical expertise required for YOLO11 implementation, which could offset the savings from platform costs. The primary cost consideration for YOLO11 implementation is human expertise rather than computational resources. Deployment requires proficiency in Linux system administration, Compute Unified Device Architecture, and CUDA Deep Neural Network library GPU driver configuration, Python programming, and deep learning frameworks such as PyTorch, typically representing senior AI and ML engineering expertise that substantially increases total implementation cost compared to the accessible web interfaces of cloud-based AutoML platforms. Our experiments used a workstation equipped with an NVIDIA Quadro RTX 5000 GPU, acquired for approximately US $3650 in 2021. The system consumes approximately 0.38 kW during training; consequently, the longest training run (approximately 13 hours) consumed an estimated 5 kWh of electricity, representing a negligible operational cost of less than US $1 at typical regional commercial electricity rates.

Among the cloud-based AutoML platforms, Vertex AI showed robustness with numerous comparative advantages, including high accuracy and *F*_1_ performance values across various datasets, with relatively fast training time and good results on different training subsets, albeit with unpredictable fluctuations and significant cost barriers (higher flat-rate pricing and continued billing even when the endpoint is idle and the object detection model is not in use), which may limit its adoption in resource-constrained health care settings.

### Limitations

The broader context of AI deployment in health care encompasses critical considerations beyond the scope of this study, including data security, privacy, and interoperability with existing hospital systems and protocols. Several limitations should be considered when interpreting our results. The dynamic model selection by cloud-based AutoML platforms prevented direct architectural comparisons, as different underlying models were selected for different dataset sizes. Edge device testing was limited to Microsoft Azure and YOLO11, as other cloud-based AutoML platforms do not support ONNX model export. Performance metrics were evaluated at a conventional confidence threshold of 0.5, which may not be optimal for all platforms or deployment scenarios. The absence of CIs and formal statistical testing across hospital sites may be viewed as a limitation. However, the cloud-based AutoML platforms evaluated do not support the cross-validation procedures necessary for consistent variance estimation, and our study design prioritized assessment of the effects of real-world human and environmental factors on model performance. Several potential sources of bias should be considered when interpreting our results. The study was limited to 30 medications representing commonly dispensed products in Hungarian clinical settings, which does not encompass all possible visual characteristics encountered across different health care contexts or geographic regions. Performance may vary on larger medication databases, different medication types, and on medications with visual characteristics underrepresented in our dataset. While our clinical dataset was designed to assess real-world deployment challenges, it may confound interpretation of site-specific performance differences, as observed performance variations reflect the combined effects of platform capabilities and local imaging conditions rather than either factor in isolation. Collection of patient-based prescription images would face substantial ethical, regulatory, and practical barriers; therefore, in the creation of our clinical dataset, we used purposive sampling with balanced class representation to enable fair cross-platform comparison. While this approach is a standard methodology for ML benchmark studies and aligns with established datasets, it does not reflect natural prescription prevalence patterns. Future implementation studies should use representative sampling within appropriate ethical frameworks to validate model performance under authentic clinical workflow conditions.

### Practical Implications

The substantial variation in performance observed across different clinical settings highlights the importance of environmental factors in model performance. The marked performance degradation observed in certain clinical settings indicates that local validation is essential before deployment.

When selecting platforms, users should consider each platform’s unique characteristics, especially when working with smaller training datasets. YOLO11 offers competitive performance without platform costs and provides superior edge device compatibility for cost-sensitive applications requiring local processing. However, larger training datasets may be necessary for optimal performance. When evaluating the total cost of ownership, YOLO11’s zero platform cost must be weighed against both hardware investment and the human capital required for implementation and ongoing maintenance after deployment. Consequently, cloud-based AutoML platforms, despite higher per-use costs, may prove more economical for some organizations and deployment scenarios. Vertex AI offers strong performance and ease of use despite higher costs, making it suitable for scenarios in which access to technical expertise is limited. Its fixed pricing model can be advantageous for situations requiring frequent processing of large volumes of images. Custom Vision and Amazon Rekognition are more economical for smaller-scale deployments, given their pricing structures. Custom Vision delivers predictable performance improvements, while Rekognition provides high-precision detection. Platform selection ultimately requires balancing technical requirements, budget constraints, and deployment scale to achieve optimal clinical pill recognition performance. For real-time point-of-care verification, response times below 2‐3 seconds generally integrate well into verification workflows without causing perceptible delays that could reduce productivity, introduce operator fatigue, or disrupt routine workflow rhythm. All prediction times observed in our study fall within this acceptable range, suggesting that computational speed is unlikely to be a limiting factor for any of the evaluated platforms.

The clinical implications of different error types warrant careful consideration for deployment planning. Missed detections and misidentifications pose a significant risk if undetected, potentially leading to missed therapeutic effects, unexpected adverse events, or dangerous drug interactions. Our findings reinforce the necessity of manual verification during clinical workflows for maintaining safety. Automated dispensing and verification systems should augment rather than replace pharmacist verification, with particular vigilance required for medications exhibiting high visual similarity to other products in the hospital formulary.

### Conclusions

This study provides the first comprehensive comparison of a traditional code-based model and cloud-based AutoML approaches for pill recognition model training intended to be used in clinical settings. While no single platform dominated across all test scenarios, each demonstrated distinct advantages for specific use cases. YOLO11 proved reliable for model development and training due to its flexibility, cost-effectiveness, and edge deployment capabilities. Vertex AI delivered a strong overall performance with minimal technical expertise required. The substantial performance differences (20.62%-91.60% difference in accuracy) observed across UCTS underscore the critical importance of imaging conditions and the value of rigorous validation for real-world applications.

Platform selection for pill recognition should consider (1) available technical expertise and resources, (2) deployment environment characteristics, (3) budget constraints, and (4) edge deployment requirements. Future research should explore performance on larger medication databases and develop standardized imaging protocols to achieve consistent results for training pill recognition systems. As both traditional deep learning frameworks and cloud-based AutoML platforms continue to evolve, periodic systematic evaluation of these technologies using existing pill image datasets could provide valuable insights into how these technologies can be used to improve medication safety through automated verification systems.

## Supplementary material

10.2196/79160 Multimedia Appendix 1 General structure of the YOLO11 model.

10.2196/79160 Multimedia Appendix 2 Evaluation metrics.

10.2196/79160 Multimedia Appendix 3 List and properties of medications included in the study.

10.2196/79160 Multimedia Appendix 4 Performance metrics of pill recognition models across different datasets.

10.2196/79160 Multimedia Appendix 5 Additional confusion matrices.

## Abbreviations

AI: artificial intelligence
API: application programming interface
AutoML: automated machine learning
AWS: Amazon Web Services
FNR: false negative rate
GPU: graphics processing unit
IoU: intersection over union
mAP: mean average precision
ML: machine learning
OER: overall error rate
ONNX: Open Neural Network Exchange
SDK: software development kit
UCTS: uncontrolled clinical test set
YOLO: You Only Look Once
