# Supplementary material for: Code-Based Versus AutoML Methods for Pill Recognition in Clinical Settings: Comparative Performance Study
Source: JMIR Med Inform. 2026 Apr 10;14:e79160. doi: 10.2196/79160 (PMC13068000; doi:10.2196/79160)
Supplement: Multimedia Appendix 1 [file medinform-v14-e79160-s001.docx]

***Multimedia Appendix 1. General structure of YOLO11 model*:**

YOLO11 introduces several key enhancements, notably the integration of the Cross-Stage Partial with Self-Attention (C2PSA) module. This module combines the advantages of cross-stage partial networks with self-attention mechanisms, enabling more effective capture of contextual information across layers. As a result, object detection performance is improved, particularly in cases involving small or occluded objects, making it the perfect choice for pill detection. Furthermore, YOLO11 replaces the C2f block with C3k2, a customized version of the CSP Bottleneck that employs two smaller convolutions instead of the single large convolution used in YOLO8. This design maintains accuracy while enhancing computational efficiency and inference speed.


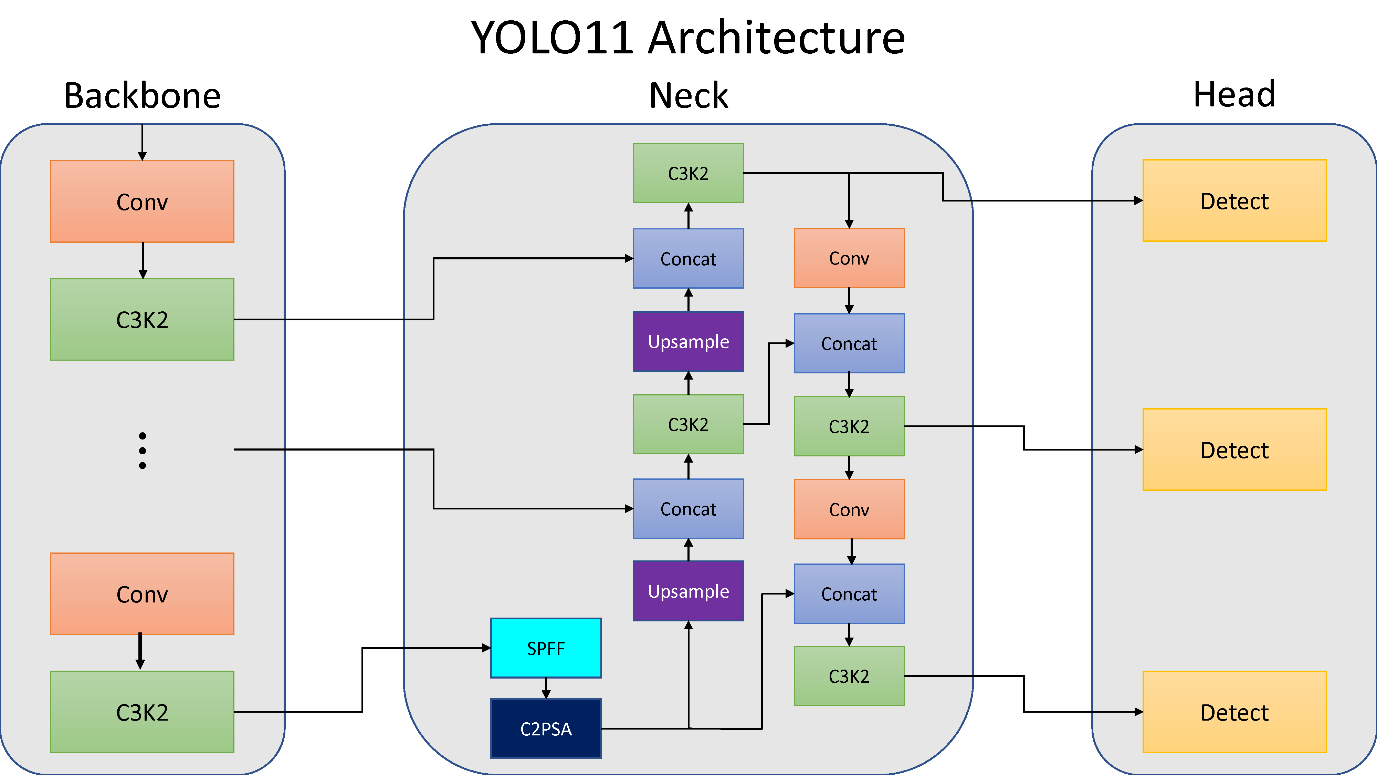
 **Supplementary Figure 1.** Generalized structure of YOLO11 model
