# Supplementary material for: Code-Based Versus AutoML Methods for Pill Recognition in Clinical Settings: Comparative Performance Study
Source: JMIR Med Inform. 2026 Apr 10;14:e79160. doi: 10.2196/79160 (PMC13068000; doi:10.2196/79160)
Supplement: Multimedia Appendix 2 [file medinform-v14-e79160-s002.docx]

**Multimedia Appendix 2. Evaluation metrics**

In object detection, models must not only classify objects but also accurately locate them. As a result, traditional image classification metrics such as precision and recall cannot be directly applied in their standard form. These metrics require adaptation to account for spatial accuracy, typically through the use of additional criteria such as intersection over union (IoU). The primary metric of choice for object detection is mean average precision (mAP). To understand the idea behind mAP, we first discuss the basics of IoU, precision, recall and the precision-recall curve.

***1. Intersection over Union***

Intersection over Union (IoU), is a fundamental metric in object detection that quantifies the overlap between predicted bounding boxes and ground-truth bounding boxes. It is defined as the ratio of intersection area to union area:

$$IoU=\frac{area of intersection}{area of union}$$

The area of *intersection* or overlap represents the common area shared by both the predicted and ground-truth bounding boxes, while the *area of union* encompasses the total area covered by both boxes combined. Ground-truth (GT) bounding boxes are typically manually annotated and represent the actual object locations, whereas predicted bounding boxes are generated by the detection model. IoU scores range from 0 to 1:

- 0.0: No overlap between predicted and ground-truth boxes
- 1.0: Perfect match, meaning the predicted box exactly fits the ground-truth one.

In practice, a prediction is often considered correct if its IoU with the GT exceeds a certain threshold, commonly 0.5. However, high-precision applications may require more stringent thresholds (e.g., 0.75 or 0.9).

In short, for pill recognition IoU measures localization accuracy for single or multiple pills.


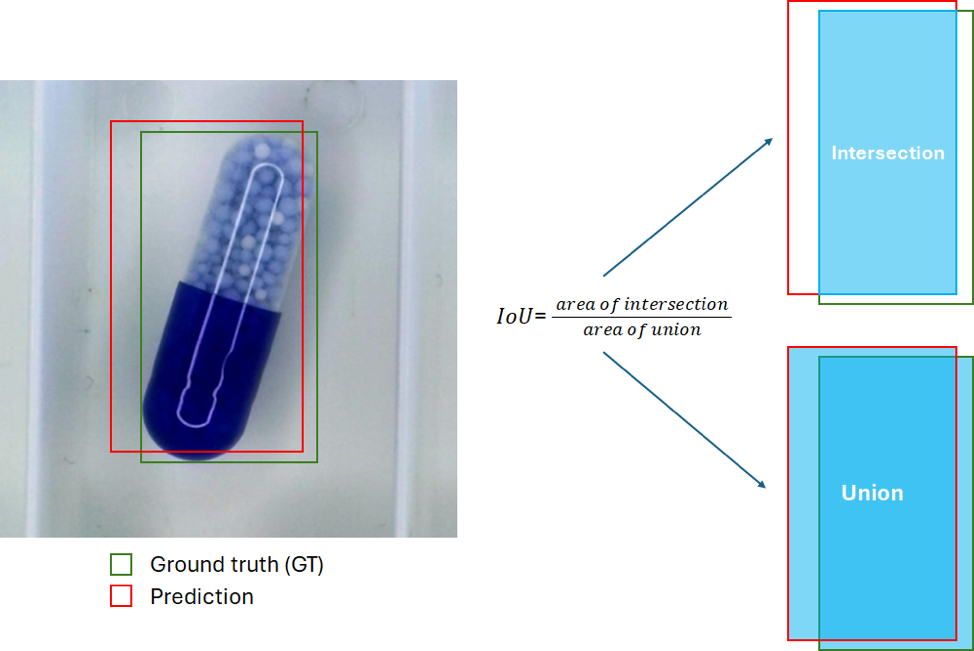


**Supplementary Figure 1.** Definition and calculation of IoU

***2. True Positives, False Positives, True Negatives, and False Negatives***

Beyond basic bounding box comparison, detailed evaluation requires understanding model behavior across scenarios. For instance, how often does the model correctly identify a bisoprolol tablet versus misclassifying it as another medication? How often are other medications mistakenly identified as bisoprolol?

Four key evaluation categories facilitate this analysis:

The confidence score in object detection reflects the model’s certainty that a predicted bounding box contains an object (often referred to as the *objectness score*) and how accurately the box aligns with the object. Ideally, if no object is present in the corresponding region of the image, the confidence score should be close to zero.

A detection is considered a True Positive (TP) if it satisfies both of the following criteria:

- The confidence score of the predicted bounding box exceeds a predefined threshold, indicating that the model believes it has detected an object of interest.
- The IoU between the predicted bounding box and the ground-truth bounding box is greater than a specified IoU threshold (typically 0.5 or higher), ensuring the predicted location closely aligns with the actual object.

A prediction is considered a False Positive (FP) under the following conditions:

- The model predicts a bounding box with high confidence, but no ground-truth object exists in that location, resulting in an IoU of zero.
- The IoU between the predicted box and the ground truth is below the IoU threshold, indicating poor localization.
- The predicted box has high IoU with the ground truth, but the predicted class label is incorrect, leading to misclassification.

A False Negative (FN) occurs when:

- An object is present in the image (i.e., there is a ground-truth bounding box),
- But the model fails to detect either because it did not predict a bounding box, or the prediction’s confidence score was below the threshold, or the predicted box's IoU with the ground truth is too low.

In object detection, True Negatives (TN) are rarely used in evaluation metrics such as Precision and Recall, as they are difficult to define consistently due to the spatial nature of detection tasks.

***3. Precision***

Precision is defined as the ratio of correctly predicted positive instances to the total number of instances that the model predicted as positive, whether correct or incorrect. Mathematically, it is expressed as:

$$\Pr ecision=\frac{TP}{TP+FP}$$

In essence, precision answers the question: *When the model predicts a sample as positive, how often is it correct?* It reflects the reliability of the model’s positive predictions.

The metric is sensitive to FPs, as these appear in the denominator. A high precision score indicates that the model makes few false positive errors, i.e., it rarely classifies negative instances as positive.

To achieve high precision, the model must maximize TPs and it must also minimize FPs. On the other hand, precision will be low if the model misclassifies negative samples as positive or it fails to correctly identify positive samples.

***4. Recall***

Recall is the ratio of correctly predicted positive instances to the total number of actual positive instances. It measures the model’s ability to identify all relevant instances of the positive class. Formally, recall is defined as:

$$Recall=\frac{TP}{TP+FN}$$

This metric answers the question: *Did the model detect all the positive cases it was supposed to?* A high recall value indicates that the model is successfully capturing most of the true positive instances.

Unlike precision, which takes into account false positives, recall is solely focused on how well the model identifies actual positives. It is independent of how negative samples are classified. Even if the model mistakenly classifies all negative samples as positive (thus inflating the number of false positives), recall can still be perfect (1.0) as long as all actual positive instances are correctly identified. Recall is maximized when the model correctly detects all true positives, minimizing false negatives. Recall drops when the model fails to identify many actual positives, resulting in a higher number of false negatives.

***5. Average Precision***

Average Precision (AP) summarizes the shape of the Precision-Recall curve into a single scalar value, providing a helpful view of how well the model balances precision and recall across varying confidence thresholds. In other words, AP is the area under the PR-curve, and it measures the overall performance of a detector model.

To make the computation of AP more consistent, an interpolated version of AP is often applied. This is referred to as the 101-point interpolated AP, as defined in the PASCAL VOC challenge. In this method the precision is interpolated at 101 equally spaced recall levels from 0 to 1. For each recall level $r$, the interpolated precision $p_{interp}\left( r \right)$ can be formulated as:

$$p_{interp}\left( r \right)=\max_{\tilde{r}\geq r} p\left( \tilde{r} \right)$$

The final Average Precision is then computed as the mean of the interpolated precisions across all 101 recall levels:

$$AP=\frac{1}{101}\sum_{\left. r\in\{0.00, 0.01,\ldots,1.00\} \right.} p_{interp}\left( r \right)$$

***6. Mean Average Precision***

While Average Precision (AP) gives a performance measure for a single class, mAP aggregates the AP values of all classes, providing a comprehensive evaluation of the model's ability to detect objects across all categories. Once we have the AP for each class, mAP is simply the mean of these AP values across all classes:

$$mAP=\frac{1}{C}\sum_{i=1}^{C} AP_{i}$$

Where $C$is the number of classes and $AP_{i}$ is the AP for the $i$-th class.

While mAP provides a general performance measure, different versions of mAP are used based on IoU (Intersection over Union) thresholds, which determine how much overlap is required for a predicted bounding box to be considered correct. These variations allow us to evaluate model performance under different levels of strictness for detection accuracy.

For instance, [mAP@0.5](mailto:mAP@0.5) measures the mean Average Precision where the IoU threshold for a correct detection is set at 0.5. This means a predicted bounding box is considered correct if its IoU with the ground truth box is greater than or equal to 0.5. The same can be applied with different threshold values. Furthermore, mAP@0.5-0.95 calculates the mean Average Precision across a range of IoU thresholds, from 0.5 to 0.95 in increments of 0.05. This version of mAP provides a more complex, more elaborate evaluation, as it evaluates performance over a range of IoU thresholds, rather than at a fixed value. It gives a better understanding of how the model behaves in different scenarios. Mathematically, we can express it as: different scenarios. Mathematically, we can express it as:

$$mAP@0.5-0.95=\frac{1}{C}\sum_{i=1}^{C} \frac{1}{N}\sum_{t=0}^{N-1} AP_{i}\left( t \right)$$

***7. Accuracy***

Accuracy is a standard metric in classification tasks, typically defined as the ratio of correct predictions (True Positives) to the total number of predictions made (True Positives + False Positives + False Negatives). In the context of image classification, accuracy simply checks if the predicted label matches the ground truth label.

However, in object detection, a simple traditionally defined accuracy is not preferred, as object detection involves not only classifying objects but also locating them within an image, i.e. predicting the correct position and bounding box for each object detected and traditional accuracy does not account for the localization aspect of the task. In object detection tasks, even if the model classifies an object correctly, the detection can still be considered incorrect if the bounding box is not well-aligned with the ground truth.

In the context of object detection, we often need a modified version of accuracy that incorporates both classification and localization. A model can be considered accurate not only if it classifies the object correctly, but also if it correctly identifies the location of the object in the image. We applied a modified version of accuracy in our experiments, where the confidence of the predicted label is not the only criterion, but the IoU between the predicted bounding box and the ground-truth bounding box is also considered to be greater than or equal to 0.5.

In this scenario, accuracy can be written as:

$$Accuracy = \frac{1}{N}\sum_{i=1}^{N} I\left( \dot{y_{i}}=y_{i} \bigwedge IoU_{i}\geq\tau\right)$$

Where:

- $N$: Total number of ground-truth objects in the dataset (or images),
- $\dot{y_{i}}$ : Predicted label for object $i$,
- $y_{i}$ : Ground truth label for object $i$,
- $I$ : Indicator function (1 if condition is true, 0 otherwise),
- $\tau$ : The IoU threshold parameter that determines how strict the localization criterion is

**8. False negative rate (FNR)**

FNR quantifies the proportion of actual positive instances, pills present in an image, that the system fails to detect. FNR is formally defined as:

$$FNR=\frac{FN}{FN+TP}$$

High FNR indicates numerous missed detections, undermining recognition system reliability. Conversely, low FNR suggests the model effectively minimizes missed detections. It answers: “Out of all the pills that were present, how many did the model fail to detect?”

FNR is crucial for patient safety, as missing a pill could result in incorrect dosages or medication identification errors.

**9. Overall Error Rate** **(OER)**

OER applies to binary or multi-class classification problems and works best with balanced datasets, where an equal number of images are present in each class.

It is defined as:

$$OER=1-accuracy$$
