# Supplementary material for: Code-Based Versus AutoML Methods for Pill Recognition in Clinical Settings: Comparative Performance Study
Source: JMIR Med Inform. 2026 Apr 10;14:e79160. doi: 10.2196/79160 (PMC13068000; doi:10.2196/79160)
Supplement: Multimedia Appendix 3 [file medinform-v14-e79160-s003.docx]

**Multimedia Appendix 3. List and properties of medications included in our study**

| **No.** | **Product name** | **Active pharmaceutical ingredient** | **Dosage form** | **Features** | **Color** | **Shape** |
| --- | --- | --- | --- | --- | --- | --- |
| 1 | Aktil Duo^®^ 875 mg/125 mg | amoxicillin; clavulanic acid | Film-coated tablet | Notch on one side, Debossed imprints on both sides | White | Oval |
| 2 | Apranax^®^ 550 mg | naproxen | Film-coated tablet | Notch on both sides | Blue | Oval |
| 3 | ASA Protect Pharmavit 100 mg | acetylsalicylic acid | Enteric-coated tablet | Smooth, Glossy, Convex | White | Round |
| 4 | Atorvox^®^ 20 mg | atorvastatin | Film-coated tablet | Smooth, Convex | White | Oval |
| 5 | Atorvox^®^ 40 mg | atorvastatin | Film-coated tablet | Smooth, Convex | White | Oval |
| 6 | Bisoprolol Sandoz^®^ 2.5 mg | bisoprolol | Film-coated tablet | Notch on one side, Debossed imprints on opposite side | White | Round |
| 7 | Bisoprolol Sandoz^®^ 5 mg | bisoprolol | Film-coated tablet | Cross-shaped notch on one side, Debossed imprints on opposite side | Yellow | Round |
| 8 | Clopidogrel Actavis 75 mg | clopidogrel | Film-coated tablet | Convex, “I” debossed on one side | Pink | Round |
| 9 | Contramal^®^ 50 mg | tramadol | Hard gelatin capsule | Opaque, Glossy | Yellow | Cylindrical |
| 10 | Controloc^®^ 40 mg | pantoprazole | Enteric-coated tablet | Convex, “P40” dark brown print on one side | Yellow | Oval |
| 11 | Coverex^®^-AS 5 mg | perindopril | Film-coated tablet | Notch on long edges, Debossed imprints on one side | Pale Green | Elongated |
| 12 | C-vitamin TEVA 500 mg | ascorbic acid | Chewable tablet | Flat | Orange | Round |
| 13 | Enterol^®^ 250 mg | lyophilized *Saccharomyces boulardii* | Hard gelatin capsule | Opaque, Glossy | White | Cylindrical |
| 14 | Espumisan^®^ Gyöngy 40 mg | simethicone | Soft gelatin capsule | Slightly opalescent, Smooth, almost spherical with a heat-seal seam. | Yellow | Oval |
| 15 | Frontin^®^ 0.25 mg | alprazolam | Uncoated tablet | Convex, Notch on one side, Debossed imprints on opposite side | White | Oval |
| 16 | Frontin^®^ 0.5 mg | alprazolam | Uncoated tablet | Convex, Notch on one side, Debossed imprints on opposite side | Pale Yellow | Oval |
| 17 | Furon 40 mg | furosemide | Uncoated tablet | Convex, Notch on one side | White | Round |
| 18 | Kaldyum^®^ 600 mg | potassium chloride | Hard gelatin capsule | Blue colored top and transparent bottom, with white and blue pellets. | Blue and White | Cylindrical |
| 19 | Kálium-R 1000 mg | potassium chloride | Uncoated tablet | Convex, Matt | White | Round |
| 20 | Magne B6^®^ | pyridoxine; magnesium lactate dihydrate | Film-coated tablet | Convex, Glossy | White | Oval |
| 21 | Magnerot^®^ | magnesium orotate | Uncoated tablet | Flat, Notch on one side | White | Round |
| 22 | Meforal^®^ 1000 mg | metformin hydrochloride | Film-coated tablet | Convex, Snap-tab on one side, Notch on opposite side | White | Elongated |
| 23 | Milgamma^®^ | benfotiamine; cyanocobalamin | Coated tablet | Convex, Glossy | Burgundy | Round |
| 24 | Nebibeta 5 mg | nebivolol | Uncoated tablet | Convex, Cross-shaped notch on one side | White | Round |
| 25 | Noacid^®^ 20 mg | pantoprazole | Enteric-coated tablet | Convex | Yellow | Oval |
| 26 | Normodipine^®^ 5 mg | amlodipine | Uncoated tablet | Convex, “5” debossed on one side | White | Elongated |
| 27 | Quamatel^®^ 20 mg | famotidine | Film-coated tablet | Convex, “F20” debossed on one side | Pink | Round |
| 28 | Quetiapine-Teva 25 mg | quetiapine | Film-coated tablet | Convex, “25” debossed on one side | Orange | Round |
| 29 | Rifamed 300 mg | rifampicin | Film-coated tablet | Convex, Matt | Burgundy | Round |
| 30 | Vitamin D3 Fresenius 1000 IU | cholecalciferol | Uncoated tablet | Flat, Notch on one side | White | Round |
