# Supplementary material for: Code-Based Versus AutoML Methods for Pill Recognition in Clinical Settings: Comparative Performance Study
Source: JMIR Med Inform. 2026 Apr 10;14:e79160. doi: 10.2196/79160 (PMC13068000; doi:10.2196/79160)
Supplement: Multimedia Appendix 4 [file medinform-v14-e79160-s004.docx]

**Multimedia Appendix 4. Performance metrics of pill recognition models across different datasets**

Note: All metrics are reported at confidence level 0.5 and IoU threshold 0.5 unless otherwise specified in column headers. Accuracy values are presented as percentages.

**Uncontrolled clinical dataset – Kaposvár**

| **YOLO11** | | | | | | | | |
| --- | --- | --- | --- | --- | --- | --- | --- | --- |
| Training Images | Accuracy (%) | Precision | Recall | OER | FNR | F1-Score | mAP@0.50 | mAP@0.50-0.95 |
| 1230 | 65.83 | 0.97 | 0.81 | 0.34 | 0.19 | 0.88 | 0.98 | 0.63 |
| 3450 | 70.21 | 1.00 | 0.85 | 0.30 | 0.15 | 0.92 | 0.99 | 0.70 |
| 7380 | 70.21 | 1.00 | 0.84 | 0.30 | 0.16 | 0.91 | 0.99 | 0.67 |
| 14,400 | 79.37 | 0.99 | 0.93 | 0.21 | 0.07 | 0.96 | 0.99 | 0.61 |
| 26,880 | 80.63 | 0.98 | 0.92 | 0.19 | 0.08 | 0.95 | 0.99 | 0.62 |
| **Google Vertex AI** | | | | | | | | |
| Training Images | Accuracy (%) | Precision | Recall | OER | FNR | F1-Score | mAP@0.50 | mAP@0.50-0.95 |
| 1230 | 69.79 | 0.92 | 0.94 | 0.30 | 0.06 | 0.93 | 0.97 | 0.53 |
| 3450 | 57.08 | 0.92 | 0.77 | 0.43 | 0.23 | 0.84 | 0.96 | 0.49 |
| 7380 | 72.29 | 0.92 | 0.94 | 0.28 | 0.06 | 0.93 | 0.94 | 0.45 |
| 14,400 | 62.08 | 0.90 | 0.93 | 0.38 | 0.07 | 0.91 | 0.94 | 0.48 |
| 26,880 | 71.04 | 0.96 | 0.90 | 0.29 | 0.10 | 0.93 | 0.97 | 0.53 |
| **Microsoft Azure Custom Vision** | | | | | | | | |
| Training Images | Accuracy (%) | Precision | Recall | OER | FNR | F1-Score | mAP@0.50 | mAP@0.50-0.95 |
| 1230 | 56.87 | 0.97 | 0.75 | 0.43 | 0.25 | 0.85 | 0.98 | 0.53 |
| 3450 | 59.79 | 0.93 | 0.79 | 0.40 | 0.21 | 0.85 | 0.96 | 0.49 |
| 7380 | 58.33 | 0.93 | 0.75 | 0.42 | 0.25 | 0.83 | 0.95 | 0.51 |
| 14,400 | 62.92 | 0.95 | 0.80 | 0.37 | 0.20 | 0.87 | 0.97 | 0.53 |
| 26,880 | 62.71 | 0.98 | 0.80 | 0.37 | 0.20 | 0.88 | 0.99 | 0.58 |
| **Amazon AWS Rekognition** | | | | | | | | |
| Training Images | Accuracy (%) | Precision | Recall | OER | FNR | F1-Score | mAP@0.50 | mAP@0.50-0.95 |
| 1230 | 55.83 | 1.00 | 0.58 | 0.44 | 0.42 | 0.74 | 0.62 | 0.34 |
| 3450 | 53.54 | 0.99 | 0.58 | 0.46 | 0.42 | 0.73 | 0.62 | 0.36 |
| 7380 | 61.67 | 0.99 | 0.67 | 0.38 | 0.33 | 0.80 | 0.69 | 0.41 |
| 14,400 | 55.21 | 1.00 | 0.61 | 0.45 | 0.39 | 0.75 | 0.65 | 0.39 |
| 26,880 | 70.63 | 1.00 | 0.76 | 0.29 | 0.24 | 0.86 | 0.76 | 0.45 |

**Uncontrolled clinical dataset – Komló**

| **YOLO11** | | | | | | | | |
| --- | --- | --- | --- | --- | --- | --- | --- | --- |
| Training Images | Accuracy (%) | Precision | Recall | OER | FNR | F1-Score | mAP@0.50 | mAP@0.50-0.95 |
| 1230 | 66.04 | 0.97 | 0.83 | 0.34 | 0.17 | 0.89 | 0.98 | 0.68 |
| 3450 | 64.17 | 0.99 | 0.85 | 0.36 | 0.15 | 0.92 | 1.00 | 0.75 |
| 7380 | 71.04 | 1.00 | 0.86 | 0.29 | 0.14 | 0.92 | 0.99 | 0.71 |
| 14,400 | 75.42 | 1.00 | 0.93 | 0.25 | 0.08 | 0.96 | 1.00 | 0.70 |
| 26,880 | 78.96 | 1.00 | 0.91 | 0.21 | 0.09 | 0.95 | 1.00 | 0.72 |
| **Google Vertex AI** | | | | | | | | |
| Training Images | Accuracy (%) | Precision | Recall | OER | FNR | F1-Score | mAP@0.50 | mAP@0.50-0.95 |
| 1230 | 89.17 | 0.98 | 1.00 | 0.11 | 0.00 | 0.99 | 0.98 | 0.59 |
| 3450 | 71.88 | 1.00 | 0.89 | 0.28 | 0.11 | 0.94 | 1.00 | 0.70 |
| 7380 | 90.62 | 0.98 | 0.99 | 0.09 | 0.01 | 0.98 | 0.98 | 0.52 |
| 14,400 | 83.13 | 0.98 | 0.98 | 0.17 | 0.02 | 0.98 | 0.99 | 0.59 |
| 26,880 | 90.00 | 1.00 | 0.96 | 0.10 | 0.04 | 0.98 | 1.00 | 0.58 |
| **Microsoft Azure Custom Vision** | | | | | | | | |
|  | | | | | | | | |
| Training Images | Accuracy (%) | Precision | Recall | OER | FNR | F1-Score | mAP@0.50 | mAP@0.50-0.95 |
| 1230 | 69.58 | 0.99 | 0.86 | 0.30 | 0.14 | 0.92 | 1.00 | 0.62 |
| 3450 | 75.21 | 0.98 | 0.92 | 0.25 | 0.08 | 0.95 | 0.98 | 0.59 |
| 7380 | 75.00 | 0.98 | 0.91 | 0.25 | 0.09 | 0.95 | 0.99 | 0.59 |
| 14,400 | 76.46 | 0.98 | 0.92 | 0.24 | 0.08 | 0.95 | 0.99 | 0.61 |
| 26,880 | 77.71 | 0.99 | 0.92 | 0.22 | 0.08 | 0.95 | 1.00 | 0.67 |
| **Amazon AWS Rekognition** | | | | | | | | |
| Training Images | Accuracy (%) | Precision | Recall | OER | FNR | F1-Score | mAP@0.50 | mAP@0.50-0.95 |
| 1230 | 68.13 | 1.00 | 0.69 | 0.32 | 0.31 | 0.82 | 0.72 | 0.45 |
| 3450 | 71.25 | 1.00 | 0.76 | 0.29 | 0.24 | 0.86 | 0.78 | 0.52 |
| 7380 | 69.17 | 1.00 | 0.71 | 0.31 | 0.29 | 0.83 | 0.71 | 0.44 |
| 14,400 | 72.50 | 1.00 | 0.75 | 0.28 | 0.25 | 0.86 | 0.76 | 0.50 |
| 26,880 | 77.71 | 1.00 | 0.80 | 0.22 | 0.20 | 0.89 | 0.79 | 0.53 |

**Uncontrolled clinical dataset – Pécs**

| **YOLO11** | | | | | | | | |
| --- | --- | --- | --- | --- | --- | --- | --- | --- |
| Training Images | Accuracy (%) | Precision | Recall | OER | FNR | F1-Score | mAP@0.50 | mAP@0.50-0.95 |
| 1230 | 22.71 | 0.93 | 0.49 | 0.77 | 0.51 | 0.64 | 0.88 | 0.68 |
| 3450 | 30.63 | 0.92 | 0.56 | 0.69 | 0.44 | 0.70 | 0.88 | 0.69 |
| 7380 | 31.67 | 0.99 | 0.56 | 0.68 | 0.44 | 0.71 | 0.85 | 0.66 |
| 14,400 | 62.50 | 1.00 | 0.89 | 0.38 | 0.11 | 0.94 | 0.98 | 0.75 |
| 26,880 | 64.58 | 0.99 | 0.91 | 0.35 | 0.09 | 0.95 | 0.99 | 0.76 |
| **Google Vertex AI** | | | | | | | | |
| Training Images | Accuracy (%) | Precision | Recall | OER | FNR | F1-Score | mAP@0.50 | mAP@0.50-0.95 |
| 1230 | 78.96 | 0.99 | 0.94 | 0.21 | 0.06 | 0.96 | 0.98 | 0.66 |
| 3450 | 70.42 | 0.99 | 0.96 | 0.30 | 0.04 | 0.97 | 0.98 | 0.70 |
| 7380 | 79.37 | 0.99 | 0.94 | 0.21 | 0.06 | 0.97 | 1.00 | 0.65 |
| 14,400 | 79.79 | 0.99 | 0.95 | 0.20 | 0.05 | 0.97 | 1.00 | 0.69 |
| 26,880 | 65.42 | 1.00 | 0.81 | 0.35 | 0.19 | 0.90 | 0.97 | 0.67 |
| **Microsoft Azure Custom Vision** | | | | | | | | |
| Training Images | Accuracy (%) | Precision | Recall | OER | FNR | F1-Score | mAP@0.50 | mAP@0.50-0.95 |
| 1230 | 49.38 | 0.98 | 0.72 | 0.51 | 0.28 | 0.83 | 0.99 | 0.62 |
| 3450 | 51.88 | 0.99 | 0.75 | 0.48 | 0.25 | 0.86 | 0.99 | 0.64 |
| 7380 | 50.83 | 0.98 | 0.73 | 0.49 | 0.27 | 0.84 | 0.99 | 0.59 |
| 14,400 | 57.50 | 0.99 | 0.78 | 0.43 | 0.22 | 0.87 | 1.00 | 0.64 |
| 26,880 | 56.04 | 0.98 | 0.81 | 0.44 | 0.19 | 0.89 | 0.99 | 0.65 |
| **Amazon AWS Rekognition** | | | | | | | | |
| Training Images | Accuracy (%) | Precision | Recall | OER | FNR | F1-Score | mAP@0.50 | mAP@0.50-0.95 |
| 1230 | 9.79 | 1.00 | 0.17 | 0.90 | 0.83 | 0.29 | 0.19 | 0.14 |
| 3450 | 17.92 | 1.00 | 0.27 | 0.82 | 0.73 | 0.43 | 0.34 | 0.24 |
| 7380 | 27.08 | 0.92 | 0.30 | 0.73 | 0.70 | 0.46 | 0.32 | 0.23 |
| 14,400 | 20.83 | 0.93 | 0.25 | 0.79 | 0.75 | 0.40 | 0.31 | 0.23 |
| 26,880 | 20.62 | 0.92 | 0.26 | 0.79 | 0.74 | 0.40 | 0.26 | 0.19 |

**Verification dataset**

| **YOLO11** | | | | | | | | |
| --- | --- | --- | --- | --- | --- | --- | --- | --- |
| Training Images | Accuracy (%) | Precision | Recall | OER | FNR | F1-Score | mAP@0.50 | mAP@0.50-0.95 |
| 1230 | 63.06 | 1.00 | 0.85 | 0.37 | 0.15 | 0.92 | 1.00 | 0.85 |
| 3450 | 67.36 | 1.00 | 0.86 | 0.33 | 0.14 | 0.93 | 1.00 | 0.86 |
| 7380 | 68.89 | 1.00 | 0.91 | 0.31 | 0.09 | 0.95 | 1.00 | 0.86 |
| 14,400 | 77.85 | 1.00 | 0.96 | 0.22 | 0.04 | 0.98 | 0.95 | 0.85 |
| 26,880 | 80.83 | 1.00 | 0.96 | 0.19 | 0.04 | 0.98 | 1.00 | 0.89 |
| **Google Vertex AI** | | | | | | | | |
| Training Images | Accuracy (%) | Precision | Recall | OER | FNR | F1-Score | mAP@0.50 | mAP@0.50-0.95 |
| 1230 | 94.37 | 1.00 | 1.00 | 0.06 | 0.00 | 1.00 | 1.00 | 0.82 |
| 3450 | 83.06 | 1.00 | 0.98 | 0.17 | 0.02 | 0.99 | 1.00 | 0.87 |
| 7380 | 94.03 | 1.00 | 0.99 | 0.06 | 0.01 | 0.99 | 1.00 | 0.81 |
| 14,400 | 85.97 | 1.00 | 0.98 | 0.14 | 0.02 | 0.99 | 1.00 | 0.81 |
| 26,880 | 91.60 | 1.00 | 0.98 | 0.08 | 0.02 | 0.99 | 1.00 | 0.79 |
| **Microsoft Azure Custom Vision** | | | | | | | | |
| Training Images | Accuracy (%) | Precision | Recall | OER | FNR | F1-Score | mAP@0.50 | mAP@0.50-0.95 |
| 1230 | 77.08 | 1.00 | 0.91 | 0.23 | 0.09 | 0.95 | 1.00 | 0.66 |
| 3450 | 79.93 | 1.00 | 0.92 | 0.20 | 0.08 | 0.96 | 1.00 | 0.68 |
| 7380 | 80.76 | 1.00 | 0.90 | 0.19 | 0.10 | 0.95 | 1.00 | 0.68 |
| 14,400 | 82.22 | 1.00 | 0.93 | 0.18 | 0.07 | 0.96 | 1.00 | 0.66 |
| 26,880 | 85.62 | 1.00 | 0.94 | 0.14 | 0.06 | 0.97 | 1.00 | 0.70 |
| **Amazon AWS Rekognition** | | | | | | | | |
| Training Images | Accuracy (%) | Precision | Recall | OER | FNR | F1-Score | mAP@0.50 | mAP@0.50-0.95 |
| 1230 | 66.74 | 1.00 | 0.68 | 0.33 | 0.32 | 0.81 | 0.72 | 0.59 |
| 3450 | 72.15 | 1.00 | 0.75 | 0.28 | 0.25 | 0.86 | 0.79 | 0.66 |
| 7380 | 81.87 | 1.00 | 0.84 | 0.18 | 0.16 | 0.91 | 0.84 | 0.71 |
| 14,400 | 79.24 | 1.00 | 0.81 | 0.21 | 0.19 | 0.89 | 0.82 | 0.68 |
| 26,880 | 84.72 | 1.00 | 0.85 | 0.15 | 0.15 | 0.92 | 0.85 | 0.71 |

**Laboratory controlled dataset - single pill images**

| **YOLO11** | | | | | | | | |
| --- | --- | --- | --- | --- | --- | --- | --- | --- |
| Training Images | Accuracy (%) | Precision | Recall | OER | FNR | F1-Score | mAP@0.50 | mAP@0.50-0.95 |
| 1230 | 30.00 | 0.99 | 0.61 | 0.70 | 0.40 | 0.75 | 0.97 | 0.66 |
| 3450 | 39.17 | 1.00 | 0.69 | 0.61 | 0.31 | 0.82 | 0.94 | 0.69 |
| 7380 | 39.29 | 0.95 | 0.71 | 0.61 | 0.29 | 0.81 | 0.93 | 0.55 |
| 14,400 | 38.33 | 1.00 | 0.88 | 0.62 | 0.12 | 0.94 | 0.96 | 0.53 |
| 26,880 | 40.00 | 1.00 | 0.87 | 0.60 | 0.13 | 0.93 | 0.96 | 0.48 |
| **Google Vertex AI** | | | | | | | | |
| Training Images | Accuracy (%) | Precision | Recall | OER | FNR | F1-Score | mAP@0.50 | mAP@0.50-0.95 |
| 1230 | 65.83 | 0.96 | 0.94 | 0.34 | 0.06 | 0.95 | 0.97 | 0.53 |
| 3450 | 46.67 | 0.91 | 0.76 | 0.53 | 0.24 | 0.83 | 0.95 | 0.49 |
| 7380 | 56.67 | 0.97 | 0.91 | 0.43 | 0.09 | 0.94 | 0.98 | 0.49 |
| 14,400 | 51.67 | 0.96 | 0.97 | 0.48 | 0.03 | 0.96 | 0.97 | 0.55 |
| 26,880 | 67.50 | 1.00 | 0.86 | 0.33 | 0.14 | 0.92 | 1.00 | 0.54 |
| **Microsoft Azure Custom Vision** | | | | | | | | |
| Training Images | Accuracy (%) | Precision | Recall | OER | FNR | F1-Score | mAP@0.50 | mAP@0.50-0.95 |
| 1230 | 38.33 | 1.00 | 0.76 | 0.62 | 0.24 | 0.86 | 1.00 | 0.57 |
| 3450 | 47.50 | 1.00 | 0.80 | 0.53 | 0.20 | 0.89 | 1.00 | 0.57 |
| 7380 | 45.83 | 0.96 | 0.75 | 0.54 | 0.25 | 0.84 | 0.99 | 0.56 |
| 14,400 | 47.50 | 0.98 | 0.75 | 0.53 | 0.25 | 0.85 | 0.99 | 0.57 |
| 26,880 | 51.67 | 0.97 | 0.78 | 0.48 | 0.22 | 0.86 | 0.98 | 0.59 |
| **Amazon AWS Rekognition** | | | | | | | | |
| Training Images | Accuracy (%) | Precision | Recall | OER | FNR | F1-Score | mAP@0.50 | mAP@0.50-0.95 |
| 1230 | 40.00 | 1.00 | 0.53 | 0.60 | 0.47 | 0.70 | 1.00 | 0.77 |
| 3450 | 46.67 | 1.00 | 0.81 | 0.53 | 0.19 | 0.89 | 1.00 | 0.72 |
| 7380 | 60.00 | 1.00 | 0.85 | 0.40 | 0.15 | 0.92 | 1.00 | 0.74 |
| 14,400 | 47.50 | 1.00 | 0.68 | 0.53 | 0.32 | 0.81 | 1.00 | 0.79 |
| 26,880 | 43.33 | 1.00 | 0.80 | 0.57 | 0.20 | 0.89 | 1.00 | 0.77 |

**Exhaustive dataset with single and multiple pills per image**

| **YOLO11** | | | | | | | | |
| --- | --- | --- | --- | --- | --- | --- | --- | --- |
| Training Images | Accuracy (%) | Precision | Recall | OER | FNR | F1-Score | mAP@0.50 | mAP@0.50-0.95 |
| 1230 | 30.66 | 0.94 | 0.54 | 0.69 | 0.46 | 0.68 | 0.85 | 0.53 |
| 3450 | 37.70 | 1.00 | 0.65 | 0.62 | 0.35 | 0.79 | 0.89 | 0.60 |
| 7380 | 26.23 | 0.97 | 0.57 | 0.74 | 0.43 | 0.72 | 0.80 | 0.42 |
| 14,400 | 39.67 | 0.99 | 0.83 | 0.60 | 0.17 | 0.90 | 0.94 | 0.46 |
| 26,880 | 44.10 | 0.99 | 0.81 | 0.56 | 0.19 | 0.89 | 0.95 | 0.40 |
| **Google Vertex AI** | | | | | | | | |
| Training Images | Accuracy (%) | Precision | Recall | OER | FNR | F1-Score | mAP@0.50 | mAP@0.50-0.95 |
| 1230 | 57.70 | 0.97 | 0.76 | 0.42 | 0.24 | 0.85 | 0.95 | 0.41 |
| 3450 | 40.16 | 0.96 | 0.59 | 0.60 | 0.41 | 0.73 | 0.90 | 0.37 |
| 7380 | 49.18 | 0.99 | 0.79 | 0.51 | 0.21 | 0.88 | 0.97 | 0.39 |
| 14,400 | 48.52 | 0.98 | 0.79 | 0.51 | 0.21 | 0.87 | 0.94 | 0.41 |
| 26,880 | 52.79 | 1.00 | 0.66 | 0.47 | 0.34 | 0.79 | 0.99 | 0.45 |
| **Microsoft Azure Custom Vision** | | | | | | | | |
| Training Images | Accuracy (%) | Precision | Recall | OER | FNR | F1-Score | mAP@0.50 | mAP@0.50-0.95 |
| 1230 | 27.87 | 0.94 | 0.51 | 0.72 | 0.49 | 0.66 | 0.79 | 0.33 |
| 3450 | 30.00 | 0.95 | 0.54 | 0.70 | 0.46 | 0.69 | 0.80 | 0.32 |
| 7380 | 29.84 | 0.94 | 0.50 | 0.70 | 0.50 | 0.66 | 0.76 | 0.31 |
| 14,400 | 31.64 | 0.92 | 0.52 | 0.68 | 0.48 | 0.66 | 0.77 | 0.32 |
| 26,880 | 33.61 | 0.92 | 0.54 | 0.66 | 0.46 | 0.68 | 0.77 | 0.33 |
| **Amazon AWS Rekognition** | | | | | | | | |
| Training Images | Accuracy (%) | Precision | Recall | OER | FNR | F1-Score | mAP@0.50 | mAP@0.50-0.95 |
| 1230 | 34.75 | 1.00 | 0.49 | 0.65 | 0.51 | 0.66 | 0.99 | 0.68 |
| 3450 | 41.80 | 1.00 | 0.74 | 0.58 | 0.26 | 0.85 | 0.99 | 0.66 |
| 7380 | 50.66 | 0.99 | 0.77 | 0.49 | 0.23 | 0.87 | 0.98 | 0.65 |
| 14,400 | 45.08 | 1.00 | 0.67 | 0.55 | 0.33 | 0.80 | 0.99 | 0.70 |
| 26,880 | 41.97 | 0.99 | 0.76 | 0.58 | 0.24 | 0.86 | 0.98 | 0.67 |
