# Supplementary material for: Code-Based Versus AutoML Methods for Pill Recognition in Clinical Settings: Comparative Performance Study
Source: JMIR Med Inform. 2026 Apr 10;14:e79160. doi: 10.2196/79160 (PMC13068000; doi:10.2196/79160)
Supplement: Multimedia Appendix 5 [file medinform-v14-e79160-s005.docx]

***Multimedia Appendix 5. Additional confusion matrices*:**

This appendix presents confusion matrices for YOLO11, Google Vertex AI AutoML Vision, and Microsoft Azure Custom Vision, complementing the Amazon Rekognition confusion matrix shown in Figure 5 of the main manuscript text. All matrices were generated using models trained on the full 26,880-image training dataset and evaluated on the verification dataset. These visualizations enable cross-platform comparison of confusion patterns and identification of medications prone to misclassification.


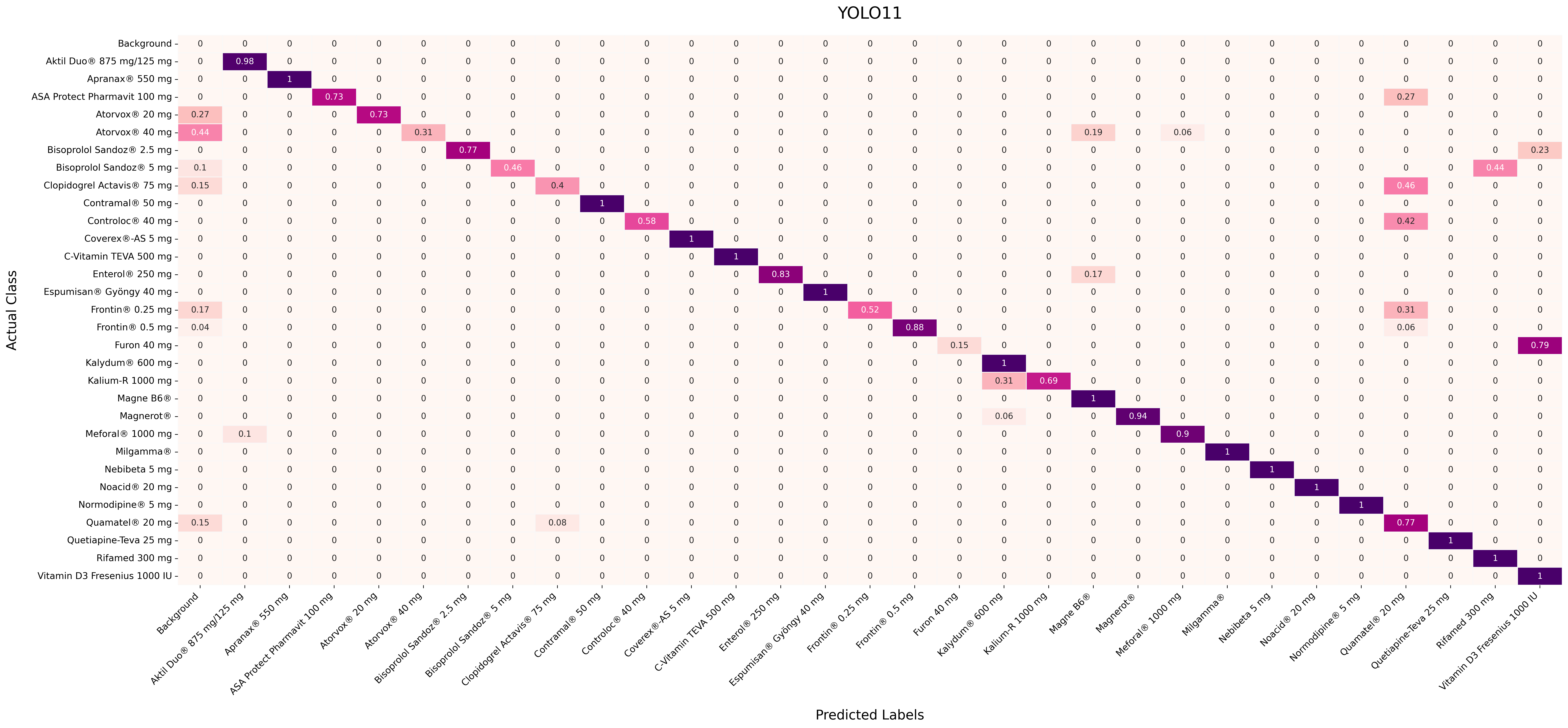


**Supplementary Figure 1.** Confusion matrix showing YOLO11 performance on the verification dataset.


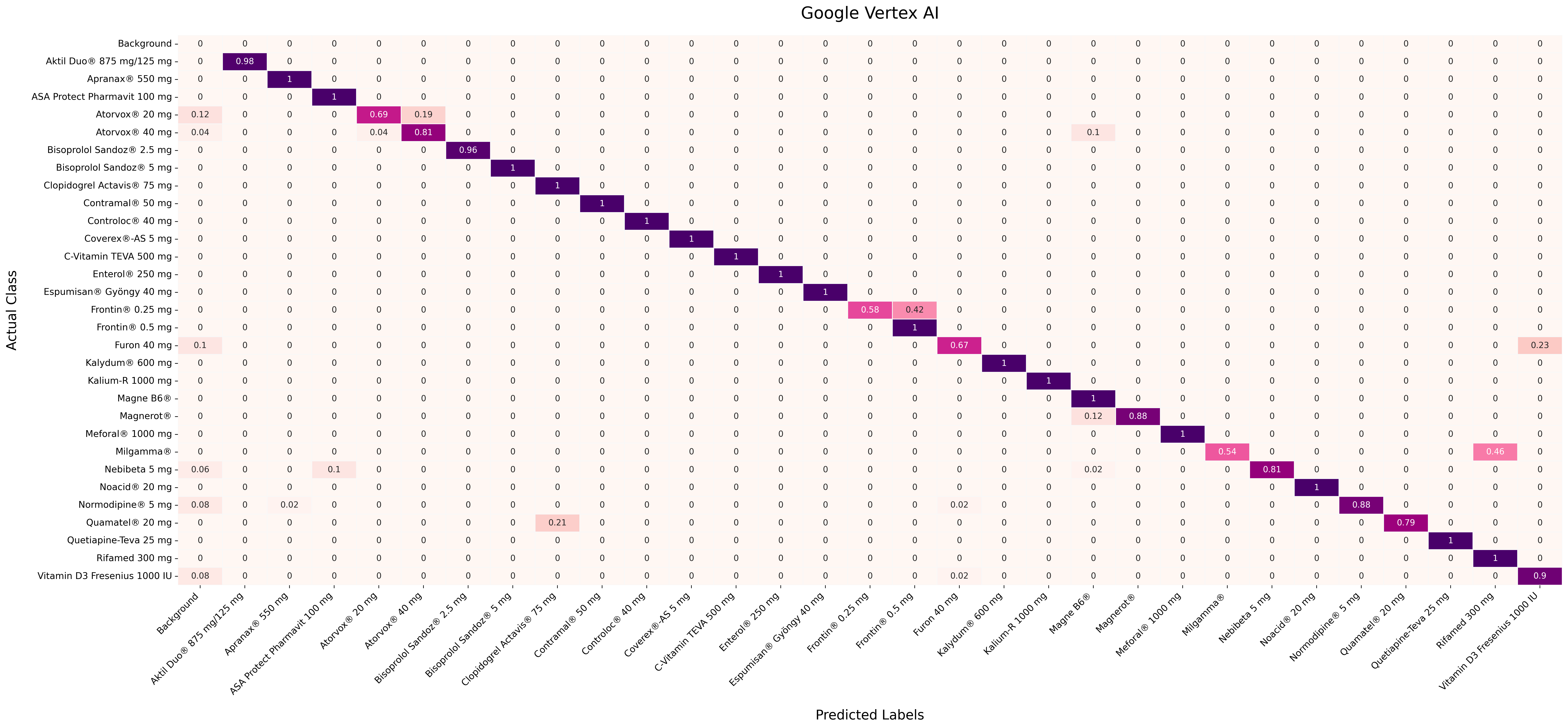


**Supplementary Figure 2.** Confusion matrix showing Google Vertex AI performance on the verification dataset.


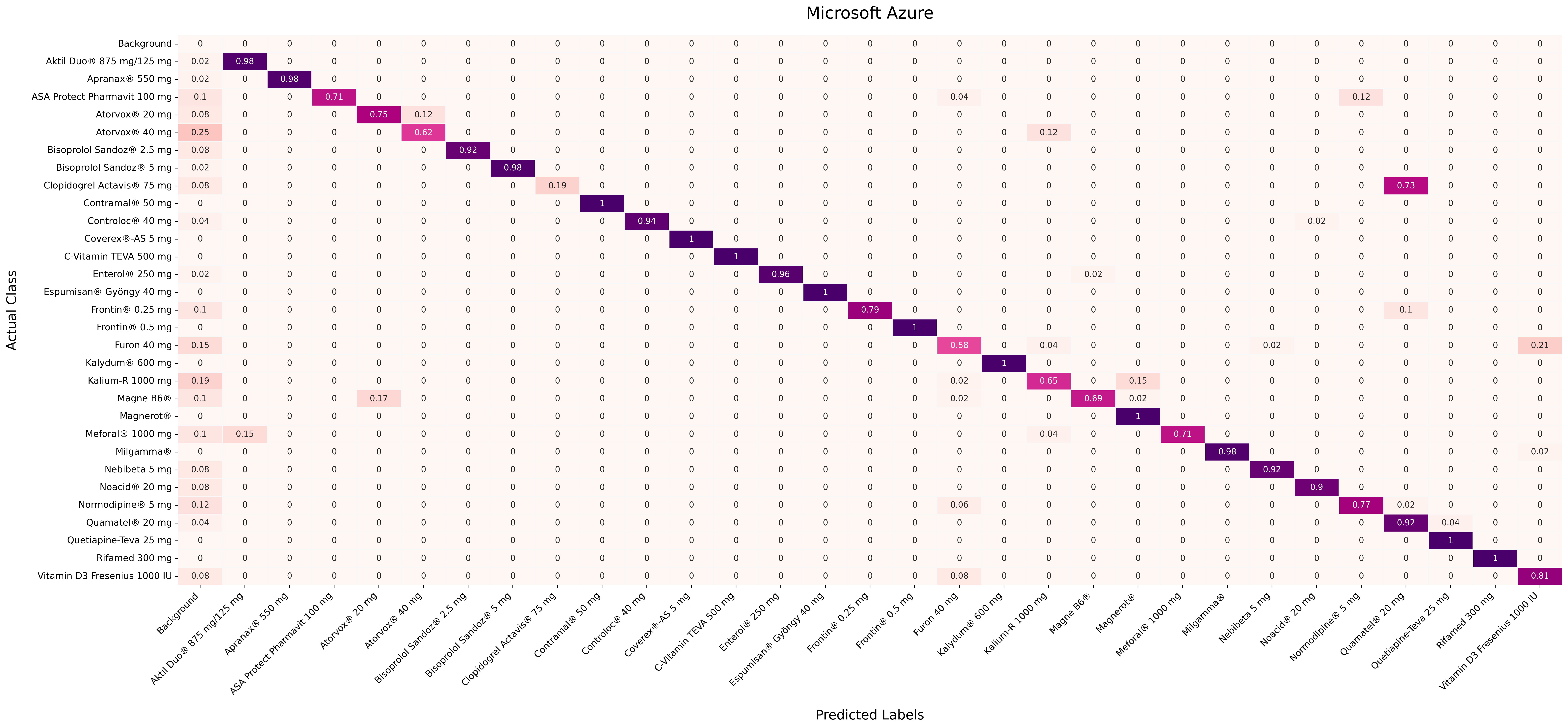


**Supplementary Figure 3.** Microsoft Azure Custom Vision performance on the verification dataset.
